# The Autocorrelated Bayesian Sampler: A Rational Process for Probability Judgments, Estimates, Confidence Intervals, Choices, Confidence Judgments, and Response Times

**DOI:** 10.1037/rev0000427

**Published:** 2023-06-08

**Authors:** Jian-Qiao Zhu, Joakim Sundh, Jake Spicer, Nick Chater, Adam N. Sanborn

**Affiliations:** 1 Department of Psychology, University of Warwick; 2 Department of Computer Science, Princeton University; 3 Department of Psychology, Uppsala University; 4 Warwick Business School, University of Warwick

**Keywords:** behavioral science, sampling, Bayesian models of cognition, normative model, rational analysis

## Abstract

Normative models of decision-making that optimally transform noisy (sensory) information into categorical decisions qualitatively mismatch human behavior. Indeed, leading computational models have only achieved high empirical corroboration by adding task-specific assumptions that deviate from normative principles. In response, we offer a Bayesian approach that implicitly produces a posterior distribution of possible answers (hypotheses) in response to sensory information. But we assume that the brain has no direct access to this posterior, but can only *sample* hypotheses according to their posterior probabilities. Accordingly, we argue that the primary problem of normative concern in decision-making is integrating stochastic *hypotheses*, rather than stochastic sensory information, to make categorical decisions. This implies that human response variability arises mainly from posterior sampling rather than sensory noise. Because human hypothesis generation is serially correlated, hypothesis samples will be autocorrelated. Guided by this new problem formulation, we develop a new process, the Autocorrelated Bayesian Sampler (ABS), which grounds autocorrelated hypothesis generation in a sophisticated sampling algorithm. The ABS provides a single mechanism that qualitatively explains many empirical effects of probability judgments, estimates, confidence intervals, choice, confidence judgments, response times, and their relationships. Our analysis demonstrates the unifying power of a perspective shift in the exploration of normative models. It also exemplifies the proposal that the “Bayesian brain” operates using samples not probabilities, and that variability in human behavior may primarily reflect computational rather than sensory noise.

Human judgment and decision-making has been studied using a wide variety of measures. Participants are asked to provide probability judgments (e.g., Costello & Watts, 2014; Dasgupta et al., 2017; Fox & Rottenstreich, 2003; Sloman et al., 2004; Zhu et al., 2020), estimates of physical quantities (e.g., Gilden et al., 1995; Jazayeri & Movshon, 2007) with associated confidence intervals (e.g., Juslin et al., 2007; Juslin & Olsson, 1997), choices (e.g., Fehr & Rangel, 2011; Tversky & Kahneman, 1974; Usher & McClelland, 2004; Wyart & Koechlin, 2016) with their associated responses times (e.g., Blurton et al., 2020; Krajbich & Rangel, 2011; Ratcliff & Rouder, 1998; Ratcliff & Starns, 2009) and confidence judgments (e.g., Baranski & Petrusic, 1998; Juslin et al., 2007; Li & Ma, 2020; Pleskac & Busemeyer, 2010). Yet while each measurement has been subject to an enormous amount of empirical and modeling work in psychology, a unified theoretical framework that can provide an integrated account of human performance across all six measures (i.e., probability judgments, estimates, confidence intervals, choices, confidence judgments, and response times [RT]) is currently lacking.

Theorists have taken steps toward a unified model from two starting points, normative and descriptive. Existing normative models are elegant, parsimonious and are easily extendable to all six measures. But these models fail to provide a satisfactory account of many important qualitative effects observed in the empirical data. By contrast, various descriptive models, which systematically deviate from normative assumptions, capture the empirical effects both qualitatively and quantitatively for up to three of these six measures, but no single model makes predictions across all measures.

Here, we develop a simple and consistent computational process that can account for a surprising variety of qualitative findings across all six measures. To achieve this goal, we build on a strong normative foundation for all six measures, rooted in a sampling approximation to Bayesian inference. This approach also implies a radical shift in viewpoint concerning the nature of the decision-making process and the origin of variability in human behavior. In the perceptual decision-making literature, existing normative models generally operate on noisy sensory information and evaluate the relative probability that this noisy information is generated by the different hypotheses (corresponding to choice options; e.g., Green & Swets, 1966; Ratcliff & Rouder, 1998). We argue, by contrast, that noise in perceptual decision-making arises primarily not through uncertainty about sensory information, but because of the inherently stochastic nature of the cognitive process that underpins Bayesian inference.

Our starting point is that exact Bayesian computations are generally intractable; and hence that a Bayesian brain can, at best, only approximate these computations. One of the most widely used approaches to such approximation in statistics and machine learning assumes that the brain draws samples from posterior probabilities, which is often very much easier than calculating those probabilities exactly. Inspired by this approach, many theorists in the Bayesian tradition have argued that the cognitive processes thus operate over these samples, rather than representations of probabilities (Dasgupta et al., 2017; Griffiths et al., 2012; Lieder et al., 2018; Sanborn & Chater, 2016; Vul et al., 2014; Zhu et al., 2020). But this process of sampling will inevitably be noisy—different samples will be drawn on different occasions. Thus, in this type of model, the main source of variability does not arise from sensory noise, but from computational noise caused by the process of sampling. In other words, instead of evaluating evidence from the sensory system or memory, we propose that the cognitive system operates on stochastically generated hypotheses.

Our aim in this article is to outline a general process that can be applied to a wide variety of measures and tasks, when equipped with a task-specific representation. Our focus is to show that this process provides a unified framework which captures a wide range of qualitative phenomena across measures and tasks, rather than to produce a comprehensive quantitative model of a particular task. The article is structured as follows. First, we review the traditional probabilistic view of normative decision-making and note its limitations in explaining psychological data. Then we propose an alternative sampling-based approximation approach to alleviate the computational burden associated with the normative models, which in turn suggests a shift in the target problem of normative concern from accumulating sensory data to integrating stochastic hypotheses. We next demonstrate the unifying power of this perspective shift by applying this rational process model, which we call the Autocorrelated Bayesian Sampler (ABS), to the six behavioral measures, emphasizing on qualitative predictions of the model. Finally, we show how to use the ABS framework to create complete cognitive models after exploring the general judgement and decision-making process in detail.

## Overview of Probabilistic Decision-Making

The idea that human decision-making process is an optimal, perhaps Bayesian, process is attractive in the light of its potential justification as the end-state of evolution and/or learning (Bogacz et al., 2006; Drugowitsch et al., 2019; Gold & Shadlen, 2002; Green & Swets, 1966; Hawkins & Heathcote, 2021; Moran, 2015; Pleskac & Busemeyer, 2010; Ratcliff & Rouder, 1998; Tickle et al., 2023). There are many task-specific Bayesian models in psychology; but in the area of cognitive and perceptual decision-making, they are often elaborations of the general decision process of signal detection theory (SDT), which describes how sensory evidence can be transformed into optimal behavior (Green & Swets, 1966).

To illustrate our discussion, we shall consider the following trial in a perceptual task as a running example: a cloud of 24 dots briefly appears on-screen (this is the stimulus, *s*).^1^ Participants might be asked to report the probability that the number of dots falls within a certain window (a, probability judgments). They may also be asked to estimate the exact number of dots (b, estimates) or to provide a confidence interval for the estimate (c, confidence interval). Alternatively, participants could be asked to decide whether the number of dots was greater or smaller than some predefined boundary (d, choices) and the experimenter might record the elapsed times for making such choices (e, RT), for example, by asking participants whether there were greater than 25 dots on the screen. The participant may also be asked to rate their confidence in their choices (f, confidence in the decision).

**Figure 1 fig1:**
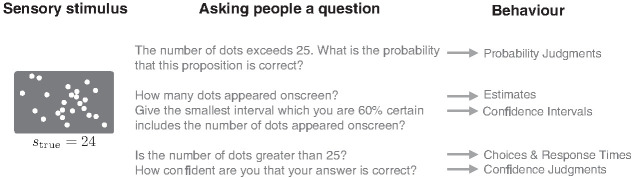
Illustrations of the Variety of Behavioral Measures for a Single Task *Note*. After the presentation of sensory stimulus, people can be asked a wide range of questions and their responses lead to corresponding behavioral measures.

Importantly, while numerosity judgment provides a concrete illustration, and one that connects naturally to existing models such as SDT, the general approach applies quite broadly. For a wide range of tasks, the six behaviors above can be collected and modeled. So, for example, participants might be asked memory-based questions about how much their last grocery bill was (e.g., “Your last grocery bill exceeded £150. What is the probability that this proposition is correct?”), or even asking participants about one-off future events such as how many years they expect to live. Thus, while we use the numerosity example in Figure 1 because it is simple and straightforward to relate to SDT, our approach applies to a wide range of cognitive and perceptual tasks, as we will see below.[Fig fig1]

More formally, in the SDT, we wish to choose between option *A* and *B*, based on a total of *T* units of sensory input (*s*_1_, *s*_2_,…, *s*_*T*_) typically assumed to be accumulated over time. Assuming that both options are equally likely a priori (i.e., *P*(*A*) = *P*(*B*)), the key variable is the summed log-likelihood ratio over the evidence from each individual unit of sensory input:

$$L(T)=\sum_{t=1}^{T}\log\frac{P(s_{t}|A)}{P(s_{t}|B)},$$ 1

where $P(s_{t}|A)$ is the likelihood of sensory evidence *s*_*t*_ when option *A* is the correct choice (similarly for $P(s_{t}|B)$). The probability of choosing *A* over *B* should be a function of the summed log-likelihood ratios. Provided with imperfect evidence (e.g., detecting a ship on a noisy radar image), SDT is a principled way to filter out irrelevant sensory noise to pick out the useful signal (e.g., whether the image contains ship). The approach can be applied in many areas of psychology, including memory, perception, and reasoning (Kellen et al., 2021; McCarley & Benjamin, 2013; Rotello, 2018; Trippas et al., 2018).

SDT, however, makes no explicit commitment on the time course of how evidence is generated and/or collected, and so makes no predictions for response time. This issue can be addressed with a dynamic extension of SDT: the sequential probability ratio test (SPRT), which postulates that the stream of sensory evidence arrives steadily and sequentially over time (Bogacz et al., 2006; Edwards, 1965; Laming, 1968). To deal optimally with the incoming sensory evidence in, for example, binary choice, the evidence should be continuously integrated into the log-likelihood ratio between the two options until a fixed threshold is reached, and RT are predicted to depend on the amount of evidence accumulated before the threshold is reached. More formally, the log-likelihood ratio for choosing option *A* over *B* is recursively updated after the arrival of each new piece of sensory evidence (*s*_*T*_):

$$L(T)=L(T-1)+\log\frac{P(s_{T}|A)}{P(s_{T}|B)}.$$ 2

**Figure 2 fig2:**
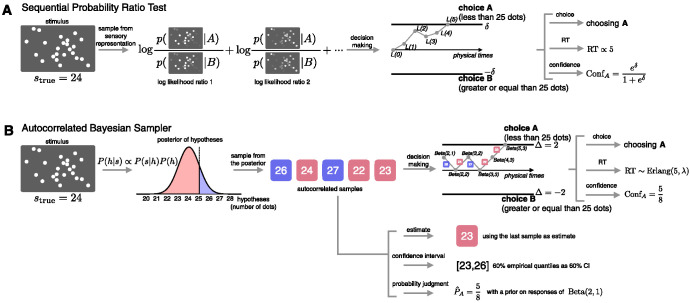
Schematic Illustrations of the Computational Mechanisms and Potential Behavioral Outputs of the SPRT (A) and the ABS (B) *Note*. A typical trial of a numerosity task is visualized in which 24 dots are briefly presented on-screen as the stimulus. The SPRT draws sequential samples from the noisy sensory representation (e.g., corrupted images), while the ABS draws autocorrelated samples of hypotheses (e.g., numbers of dots). SPRT = sequential probability ratio test; ABS = Autocorrelated Bayesian Sampler; RT = response times. See the online article for the color version of this figure.

Once the log-likelihood ratio reaches a threshold (assuming symmetric thresholds: *L*(*T*) > δ or *L*(*T*) > −δ), the evidence accumulation process stops and the response depends on whether the positive or negative threshold is reached. Increasing the magnitude of the threshold (δ) produces a slower but more accurate response as more sensory evidence, on average, is accumulated before either threshold is reached. The SPRT is optimal in the sense that the expected amount of evidence (i.e., *T*) is minimized for any fixed probability of deciding incorrectly (Wald & Wolfowitz, 1948). In other words, following the SPRT allows for the fastest response time for a particular error rate. Because the sensory inputs are assumed to be independent of one another, the SPRT is a random walk model whose starting point is *L*(0) = 0 and with two absorbing states: −δ and δ (see Figure 2A).[Fig fig2]

While intuitive and simple, the framework of the SPRT also makes decisions that take time, as people do, which is an advantage over SDT in modeling empirical data. Indeed, the SPRT can produce human-like speed–accuracy tradeoffs: requiring faster decisions reduces accuracy, while requiring more accurate decisions reduces speed. This is captured in the model by assuming that people control the magnitude of the thresholds to suit their objectives. In response to an experimental emphasis on speed (accuracy), people are assumed to decrease (increase) the decision threshold; the model’s guarantee of optimal performance implies that these two measures will trade off against one another.

Unfortunately, the SPRT does not easily explain other psychological relationships between choice and RT. In binary choice, for example, the SPRT predicts identical response time distributions for choosing either of the two options (assuming an unbiased starting point, *L*(0) = 0, and symmetric thresholds), contradicting the empirical observation that mean RTs differ for correct and incorrect decisions (Ratcliff & Rouder, 1998; Stone, 1960). This is far from the only issue: Table 1 summarizes several qualitative effects of choice, response time, and confidence, the majority of which cannot be accommodated by the SPRT.[Table tbl1]

These stylized facts have been used to motivate descriptive models, including the family of models known as drift diffusion models (DDMs), that relax the normative SPRT framework to better describe human data, specifically regarding three key measures: choice, response time, and confidence. While such approaches have been highly successful, our focus here remains on approaches closely tied to normative depictions of behavior, though we return to DDMs and other common descriptive models below.

## A Representation for Producing Estimates and Confidence Intervals

The categorical-hypotheses representations used in SPRT can produce choice, response time, and confidence measures, but are not fine-grained enough to produce probability judgments (e.g., judge the probability that the number of dots was greater than 25), estimates (e.g., how many dots are there on the screen), or confidence intervals (e.g., placing a 95% confidence interval around the estimate). What is needed is an extension of the hypothesis space beyond the categorical hypotheses used when making a choice. In principle, within a Bayesian framework, this is straightforward, although the resulting model looks very different. Instead of simply using two categorical hypotheses (e.g., whether there are more than 25 dots on the screen), the model can instead represent the fine-grained hypotheses relevant for estimates (e.g., with a hypothesis corresponding to each of exact number of dots on the screen). With such a representation, estimates and confidence intervals can simply be a function (e.g., the mean and quantiles respectively) of this distribution. The probabilities of categorical hypotheses used to produce choices, confidence judgments, and RT can be calculated simply by summing up the posterior probability of the fine-grained hypotheses that are consistent with each choice (e.g., summing the probability of all the hypotheses in which the number of dots is more than 25).

This representational change, however, does not allow a probabilistic model to account for many of the empirical effects found with estimates and confidence intervals. For estimates, anchoring effects demonstrate a dependence on preceding choices even when the choice question that provides an “anchor” transparently contains no information (Tversky & Kahneman, 1974). Moreover, in the empirical data, estimated confidence intervals are typically far too narrow and are strikingly different depending on whether participants produce or evaluate them (Juslin et al., 2007). In addition, a long line of empirical work shows that probability judgments are systematically biased and incoherent (e.g., subadditivity, conjunction fallacies, partition dependence), which seems to argue against *all* purely probabilistic models (e.g., Dasgupta et al., 2017; Tentori et al., 2013; Tversky & Kahneman, 1983; Tversky & Koehler, 1994; Zhu et al., 2020).

Exact probabilistic models also show fundamental mismatches with the results of recent investigations into the source of noise in human judgment and decision-making. While probabilistic models assume a noise-free inference process using precise probabilities, there is growing empirical evidence suggesting that much, or even most, variability in decision-making in fact arises from “computational noise” (i.e., variability in precision and approximation used to perform inference) rather than “sensory noise” (i.e., variability in relevant sensory features) or “decision noise” (i.e., variability associated with action selection; Drugowitsch et al., 2016; Findling & Wyart, 2021; Stengård & van den Berg, 2019; Wyart & Koechlin, 2016). Clearly, then, there are problems with the descriptive adequacy of all probabilistic models, including SDT and the SPRT, which may stem from the psychologically implausible assumption of exact calculation of probabilities and the lack of mechanism to account for the stochasticity in the inference process. In the next section, we propose how to address these fundamental problems, before evaluating how far the proposed solution produces a better qualitative match to a wide range of regularities in human behavior.

## A Sampling-Based Approximation Perspective for Rational Decision-Making

Assuming imprecise probabilities does not necessarily mean abandoning probabilistic models. While exact Bayesian computation is often out of reach for real-world computational mechanisms, including the human brain (Anderson, 1991; Aragones et al., 2005; Kwisthout et al., 2011), computer scientists and statisticians have devised a number of sophisticated, general-purpose approximations for producing useful answers with a more reasonable amount of computational time and effort. It is therefore interesting to explore whether the brain has hit on similar solutions. One major family of general-purpose approximations in computer science and statistics is based on sampling.^2^

Following the Bayesian approach, we propose that people solve cognitive tasks by building an internal model and posterior distribution over fine-grained hypotheses, which can support the responses for all of the aforementioned six behavioral measures. But, because the exact representation of the posterior probabilities of hypotheses is typically computationally intractable, we further hypothesize that the posterior probability distribution is not computed exactly, but is approximated by drawing representative samples from the posterior. Sampling-based approximations to the posterior are appealing as a psychological mechanism because (a) some sampling algorithms (e.g., Markov Chain Monte Carlo [MCMC]; Brooks et al., 2011) need only local knowledge of the target posterior distribution and can represent only one or a few hypotheses at a time, lending these algorithms psychological plausibility (Anderson, 1991; Sanborn et al., 2010), (b) sampling algorithms show much of the same behavioral variability and deviations from ideal probabilistic inference as observed in people across a range of domains (Dasgupta et al., 2017; Griffiths et al., 2012; Lieder et al., 2018; Sanborn & Chater, 2016; Vul et al., 2014; Zhu, Newall, et al., 2022), and (c) the variability of sampling algorithms has been found to match neural variability in the cortex (Fiser et al., 2010; Haefner et al., 2016; Hoyer & Hyvärinen, 2003). These observations suggest that the sampling-based explanations can connect with all three of Marr’s (1982) celebrated explanatory levels: computational (through implementing Bayesian inference), algorithmic (via a tractable computational mechanism), and implementational (through potentially mapping on to neural activity).

Taking a sampling-based approximation perspective to model choices suggests decision-making should be conceptualized as the problem of integrating a sequence of stochastic *hypotheses* into categorical decisions. The key distinction with other probabilistic models such as SDT and the SPRT is that we specifically define the “evidence” as samples of hypotheses, abstracting away from noisy sensory percepts or memory traces used in previous models, including computational-level models (see Figure 2B). This implies that it is computational noise in the inference process that is the primary source of variability in behavior.

## The Autocorrelated Bayesian Sampler

Here we outline a rational process for producing probability judgments, estimates, confidence intervals, choices, confidence judgments, and RT based on a sampling approximation of the posterior probability of fine-grained hypotheses, which we call the ABS. Our key theoretical contribution is to create links between the sampling process and each of the six behavioral measures. This is possible because samples of the fine-grained hypotheses contain all of the relevant information to produce these (and indeed many other) aspects of behaviors.

Continuing our numerosity example (see Figures 1 and 2), the ABS produces behavior based on the posterior probability of the hypotheses, P(h|s), which is calculated using Bayes rule:

$$P(h|s)=\frac{P(s|h)P(h)}{P(s)},$$ 3

where *h* is a hypothesis, *s* is a stimulus, P(s|h) is the likelihood of a stimulus given a hypothesis, *P*(*h*) is the prior probability of a hypothesis,^3^ and *P*(*s*) is the overall probability of observing the stimuli across all possible hypotheses included in the internal model. In the numerosity task, for example, the hypothesis space reflects all possible numbers of dots that may have appeared on-screen, while the posterior distribution could be represented as a Gaussian distribution with mean equal to 24—the true number of dots. The variance of the distribution may stem from all kinds of uncertainties including, for example, perceptual noise and/or uncertainties associated with the processing of information.

This general framework applies far beyond numerosity, of course. For example, it can be applied to intuitive physics when *h* is a complete object trajectory and *s* is the initial movement of an object (e.g., Battaglia et al., 2013; Hamrick et al., 2015; Sanborn et al., 2013), language production when *h* is the next word in a sentence and *s* are the preceding words (e.g., Chater & Manning, 2006; Levy et al., 2008), and common-sense reasoning about other minds when *h* is a social goal of other agents and *s* is a sequence of actions performed by those agents (e.g., Baker et al., 2008, 2009). Similarly, Bayesian models have also been successfully implemented in explaining effects in other areas of psychology such as vision (e.g., Yuille & Kersten, 2006), motor control (Körding & Wolpert, 2004), causal reasoning (e.g., Abbott & Griffiths, 2011; Bramley et al., 2017), reading (Norris, 2006), and learning (e.g., Courville & Daw, 2007; Gershman et al., 2010).

Next, a set of hypotheses is sampled from the fine-grained posterior distribution, and these samples directly and straightforwardly support all six of our measures (Figure 2B). Probability judgments are based on the relative proportion of the samples (e.g., the number of samples with numerosities greater than 25). Estimates are based on a summary statistic of the samples (e.g., the mean sampled numerosity or the value of the most recent sample). Confidence intervals are based on the quantiles of the samples (e.g., ordering five samples and using the numerosities of the second and fourth sample as the bounds of a 60% confidence interval). Choices are based on the preponderance of the samples (e.g., depending on whether more than half the samples have numerosities greater than 25). Confidence judgments are (like probability judgments) based on the relative proportion of the samples that agree with the choice. RT are a function of the number of samples drawn (e.g., assuming that on average drawing four samples takes longer than three).

To generate concrete predictions from the model, and assess the match with human behavior, we need to outline three further aspects of the model: the choices of *sampling algorithm*, *prior on responses*, and *stopping rule*, to which we now turn.

### The Sampling Algorithm

We assume that the mind conducts sampling-based approximations by drawing samples of hypotheses in proportion to the posterior probabilities associated with each hypothesis (e.g., Chater et al., 2020; Dasgupta et al., 2017; Griffiths et al., 2012; Vul et al., 2014; Zhu et al., 2020). Rather than reviewing the extensive literature on sampling algorithms in statistics and computer science (see Andrieu et al., 2003, for an overview), we focus on algorithms that have been previously shown to match human behavior in some area of psychology.

The simplest sampling algorithm is direct sampling, in which independent and identically distributed (i.i.d.) samples are drawn (e.g., Vul et al., 2014). However, a lot must be known about the target distribution to draw i.i.d. samples: people need (at least implicitly) to know the posterior probability of every hypothesis, which fails to alleviate the computational intractability problem that motivates the need for sampling-based approximations. Another difficulty for direct sampling is descriptive. Human hypothesis generation is not a process of drawing independent samples, as direct sampling requires. Instead, what comes to mind now depends on what came to mind in the past (Dasgupta et al., 2017; Gilden et al., 1995; Zhu, León-Villagrá, et al., 2022).

In light of this, researchers have recently started to explore a family of more sophisticated sampling algorithms called MCMC (Robert & Casella, 2004). MCMC algorithms explore the hypothesis space using only local knowledge about the probability distribution, greatly reducing the knowledge required to generate samples. The key idea of MCMC is that, in its simplest form, it represents only a single hypothesis at a time, and probabilistically transitions between hypotheses in proportion to their posterior probabilities. The local transitions induce a serial dependence between samples, akin to the local transitions in human hypothesis generation (Bramley et al., 2017; Fränken et al., 2021).

In our own work, we have found that an extension of MCMC, named MC^3^, provides a close match to the dynamics of repeated human judgment, capturing the observed long-range autocorrelations between estimates, as well as the heavy-tailed distribution of absolute differences between successive estimates (Spicer et al., 2022b; Zhu et al., 2019, 2021; Zhu, León-Villagrá, et al., 2022). We therefore use MC^3^ as the sampling algorithm in the present model, though the specific mechanics of this algorithm beyond dependencies between samples are not necessary for almost all of the behaviors targeted here (see Appendix A, for algorithmic details). In other words, with the exception of explaining the cross-trial autocorrelation results which requires quantitative characterizations of the dependence in samples, the key condition for a sampler to reproduce the qualitative model behaviors (e.g., comparing average model behaviors between experimental conditions) is simply that sampling is local and autocorrelated. Thus, most model predictions can be replicated using many other MCMC sampling algorithms, including the widely used Random Walk Metropolis algorithm, so long as samples are positively correlated across time.

Using dependent samples influenced our choice for how the ABS produces estimates. In past work, estimates have been based on the most recently generated sample or by averaging over samples (Lieder et al., 2018; Vul et al., 2014). While the mean of a set of independent samples is clearly a better estimate of the underlying mean than a single sample, with dependent samples, earlier samples are more likely to be biased by the starting point than later samples. For this reason, we chose to use the most recent sample as our estimate. However, these two approaches do not predict qualitatively different behavior in aggregate (see Appendix E, for details).^4^

Producing confidence intervals, however, requires more than a single sample, and instead can reflect statistics of the entire set of samples: for example, the 2.5% and 97.5% quantiles of the samples can represent a 95% confidence interval of the target distribution. This approach can only be applied directly for large samples. With small samples, we produce more fine-grained intervals by following Juslin et al. (2007) and use linear interpolation to fill in the gap between the two quantiles of the samples.

We also assume that sampling takes time. For simplicity, we model the time necessary to produce *N* samples as a Poisson process: while time taken to produce a new sample is random, the samples are generated at a constant rate (λ samples per sec). In a Poisson process, the waiting time between samples is exponentially distributed, and the time necessary to generate *N* samples follows an Erlang distribution:

f(t)∼Erlang(N, λ). 4

The mean and variance of RT for a sample size of *N* are $E[t]=\frac{N}{\lambda }$ and $V[t]=\frac{N}{\lambda^{2}}$ respectively. Using a Poisson process allows us to more closely link our approach to existing models such as the Poisson random walk (PRW) model (Blurton et al., 2020; discussed below), though the results in this article would be qualitatively the same under a wide variety of assumptions of how long it takes to generate each additional sample. This is because many empirical results only require assuming the samples were generated sequentially and generating more samples typically takes more time. Exponential waiting times are assumed here to explain the shape of RT distributions, particularly the observation that the RT for probability judgments (which we assume to have been produced using a fixed number of samples) are positively skewed (see Appendix F).^5^

### The Bayesian Monte Carlo Prior on Responses

Samples of the fine-grained hypotheses generated from our sampling algorithm can be readily used to make a choice. In our numerosity example, if asked to decide whether the number of dots that appeared on-screen is greater than 25, the hypothesis space should be partitioned into two subspaces with 25 on the boundary line. Samples that indicate greater than 25 dots or not support the corresponding hypotheses. In other words, evidence is directly translated from the samples, here taking one of two values. And also, unlike the evidence used in SPRT, there is no inherent uncertainty about which alternative each sample supports. For the numerosity example, the generated sample can denote any number of dots in the hypothesis space, but it can only support one alternative in decisions: if the sample was 23, it only supports the hypothesis that there were less than 25 dots on screen. Similarly for *M*-alternative choices (*M* > 2), the hypothesis space can be partitioned into *M* subspaces with hypothesis samples from each subspace supporting the corresponding alternative.

These samples implicitly carry information about the probability that each choice alternative is correct. For example, when asked about the probability that the number of dots is greater than 25, the relative frequency of evidence in favor of, rather than against, the event should inform the probability estimate. But, as explored in Zhu et al. (2020), people should not directly use the relative frequency of the hypotheses as a probability estimate. This is especially true when sample sizes are small because the relative frequency tends to be extreme. Indeed, a single sample would lead to a probability estimate of either 0 or 1. This problem can be solved by incorporating a prior on responses to temper the relative frequency in the estimates of the probability that each choice alternative is correct, an approach that in statistics is called Bayesian Monte Carlo (Gelman et al., 2013; Rasmussen & Ghahramani, 2002).^6^

The Bayesian Sampler model of Zhu et al. (2020) used a fixed prior on responses, and for mathematical simplicity, this was chosen to be a Beta distribution, because this is the conjugate prior for probability estimates. The Beta distribution is bounded by 0 and 1, and has two parameters, α_0_ and α_1_, which determine its shape: when both parameters exceed 1, the Beta distribution is unimodal with a peak in the middle of the range (i.e., at $\frac{\alpha_{1}-1}{\alpha_{0}+\alpha_{1}-2}$); when both parameters equal 1, it is uniform; and when both parameters are less than 1, it is bimodal with peaks at both 0 and 1. Most critically, using the Beta distribution as the prior enables evidence to act as pseudocounts in the parameters. For *S*(*A*) pieces of evidence of event *A*, *S*(¬*A*) of event not-*A*, and a Beta(α_0_,α_1_) prior, people will have a posterior distribution for probability estimates that is distributed according to Beta(α_0_ + *S*(*A*), α_1_ + *S*(¬*A*)). The Bayesian Sampler model used the expected value of this posterior distribution as its probability estimate, which is also simple to calculate:

$$\hat{P}(A)=\frac{\alpha_{0}+S(A)}{\alpha_{0}+S(A)+\alpha_{1}+S(\neg A)}=\frac{\alpha_{0}+S(A)}{N+\alpha_{0}+\alpha_{1}},$$ 5

where *N* = *S*(*A*) + *S*(¬*A*) denotes the total number of samples that were generated and translated into evidence. Both the prior parameters (which affect α_0_, α_1_) and the sampling process (which affects *S*(*A*) and *N*) affect the expected value. As the prior parameters are defined to be nonnegative (i.e., α_0_, α_1_ ≥ 0), the Bayesian Sampler’s estimated probabilities tend to avoid extreme values and regress to the mean of the prior (i.e., α_0_/(α_0_ + α_1_)).

Here, we generalize the prior on responses used in the original Bayesian Sampler in two ways. The first is to make it multivariate: in many situations, people can be asked to judge a multivariate event where the hypothesis space should be partitioned into many subspaces. For example, when asked “what is the probability that the hottest day of the week will be Sunday?,” there are seven options to consider (“Sunday hottest,” “Monday hottest,” and so on). In this case, the Dirichlet distribution, a multivariate generalization of the Beta distribution, is the natural conjugate prior. For an *M*-variate Dirichlet prior, Dir(**α**), with **α** = (α_0_, α_1_,…, α_*M*−1_), people report the mean posterior distribution as their probability estimate:

$$\hat{P}(A)=\frac{\alpha_{0}+S(A)}{N+\sum_{m=0}^{M-1}\alpha_{m}}.$$ 6

**Figure 3 fig3:**
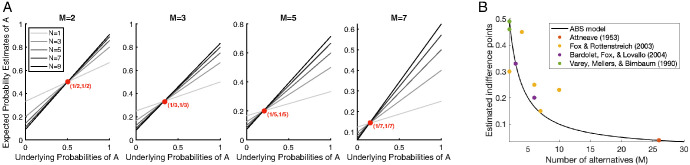
Relationship Between the Underlying Probabilities of Event A and the Average Probability Judgments of A Predicted by the ABS *Note*. (A) From left to right, the number of alternatives, *M*, varies from 2 to 7. Within each panel, simulated sample sizes, *N*, range from 1 to 9 in increments of 2. While uniform Dirichlet priors were used in the simulation, the indifferent points are always located at (1/*M*, 1/*M*) for symmetric Dirichlet priors. (B) As produced by the ABS, the empirical indifference points between mean probability judgments and objective probabilities are related to the inverse of the number of alternatives (*M*). Indifference points were directly reported by the data analyses in Fox and Rottenstreich (2003), Bardolet et al. (2011), and Varey et al. (1990), and were inferred from the regression in Attneave (1953). ABS = Autocorrelated Bayesian Sampler. See the online article for the color version of this figure.

This view of probability estimates implies an indifference point (when the underlying probability and the estimated probability matches) that depends on the number of alternatives (see Figure 3A).[Fig fig3]

The second way in which we generalize the prior on responses of the Bayesian Sampler is to allow it to adapt to experience (e.g., the trial history in an experiment). In Bayesian data analysis, when no prior information is available, a default prior is typically recommended (Gelman et al., 2013). However, for many real-world applications and especially for everyday cognitive tasks, historical data (e.g., past experiences of the same task, data from previous similar tasks or from observing others’ performing the task) are available which can help people can construct an appropriate prior. For example, if repeatedly choosing between the same two alternatives, historical choice data should provide useful information such as the base rate, which in turn can help construct a prior on responses to guide future decisions. How to construct an adaptive prior based on historical data is a topic of debate in statistics and computer science because it is difficult to determine how much to generalize previous experience to new situations (Chen et al., 2000; Diaconis & Ylvisaker, 1979; Ibrahim et al., 2015).^7^ For simplicity, we assume that people only use information from the immediately previous trial to develop their adaptive prior for the present trial: in binary choice, a noninformative, uniform prior (Beta(1,1)) is adjusted to favor the option the feedback indicated was correct, thus becoming either Beta(2,1) or Beta(1,2).

The adaptive prior on responses, in conjunction with the generated samples, then determines the model’s estimated probability of a categorical alternative being correct. This estimated probability is used both as the model’s probability estimate *and* its confidence judgment in whether a choice is correct.^8^ The equivalence between the two is not unique to our model—it has been previously posited as the Bayesian Confidence Hypothesis (Kepecs & Mainen, 2012; Mamassian, 2016; Pouget et al., 2016), and has attracted both support (Calder-Travis et al., 2020) and criticism (Li & Ma, 2020).

### The Stopping Rule

Any model of judgment or decision that depends on the sequential accumulation of evidence needs a rule determining at what point to stop collecting evidence and make a decision. When to stop drawing samples should depend on both the costs (e.g., metabolic, opportunity, etc.) of sampling as well as the task-specific benefits of additional samples for providing a good response.

**Figure F1 fig16:**
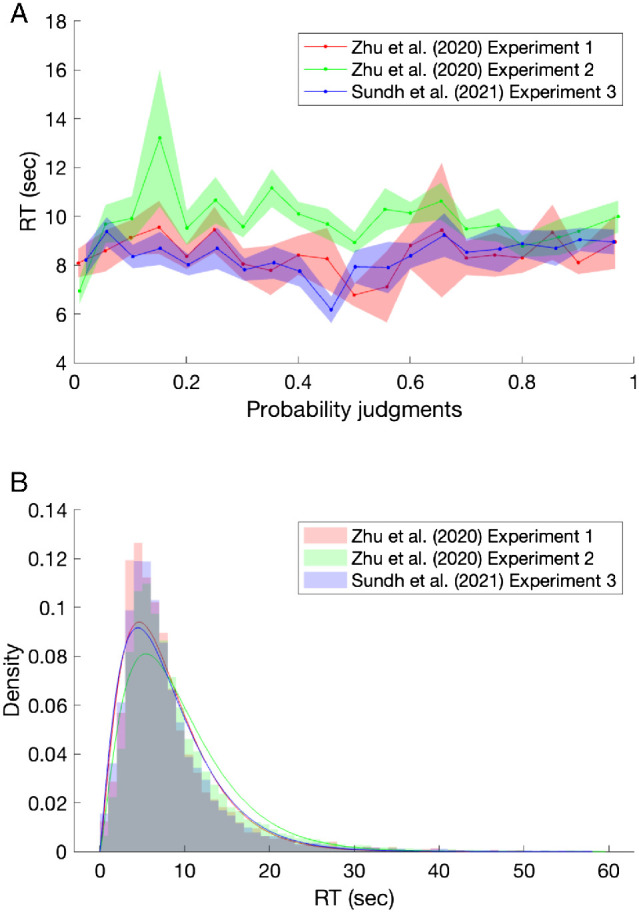
Probability judgments and Response Times *Note*. (A) Window-binned (bin width equals to 0.05) probability judgments show no relationship with response times. Empirical data were reanalyzed from the three experiments shown in the legend. Dots are mean RTs and shaded areas cover 95% confidence interval. (B) Histograms of RT data. Across the three experiments, RTs for probability judgments are unimodal and positively skewed. Colored lines are best-fitting Gamma distributions. RTs greater than 60 s were excluded from analysis. RT = response times. See the online article for the color version of this figure.

For probability judgments, estimates, and confidence intervals, in the absence of a clear alternative stopping rule, we make the simplest possible assumption: that a fixed number of samples are drawn to answer each question (though we revisit this point in Appendix F). A fixed number of samples will allow the model to produce indifferent probability judgments (e.g., judging a binary event to have a probability of 0.5) as is often observed in the human data,^9^ and fits with the even distribution across reaction times observed in our own experiments (see Figure F1 in Appendix F).

However, for making decisions, rather than probability judgments, a fixed sample is likely to be too simple. If the samples so far leave the evidence finely balanced regarding which decision to make, then it is likely that more data will be collected. While it is possible in principle to derive an optimal stopping rule for the sampling process in this model, unlike with the SPRT, the optimal rule is not analytically tractable and can instead only be computed using dynamic programming (see Appendix C). So, again for simplicity, we use a well-known heuristic stopping rule instead: the max-minus-next rule, which counts the difference in evidence between the top two hypotheses, and terminates the sampling process whenever the accumulated difference exceeds a threshold. This simple heuristic stopping rule has also been shown to approach the performance of an optimal SPRT even in multi-alternative settings (Dragalin et al., 1999, 2000). For binary choices, this reduces to just the difference in the number of samples in favor of each alternative, which has been proposed in past work (Hamrick et al., 2015; Vul et al., 2014). The decision-making panels of Figure 2B demonstrates the max-minus-next stopping rule with a threshold value of 2.

While the choice of stopping rule does not change how samples are used to produce the different measures, it does influence the content of the samples and the variability of the sample size and hence responses times. So, for example, in our model, while the RT for a *probability judgment* which is assumed to have a fixed sample sizes will follow an Erlang distribution (see Appendix F, for further justification), RT for a *choice* (which assumes optional stopping) will follow a mixture of Erlang distributions (see Appendix B, for details).

## Explaining Key Empirical Targets in Probability Judgments, Estimates, Confidence Intervals, Choices, Confidence Judgments, and RT

We now demonstrate the explanatory power of the ABS. We focus on behavioral results that deviate from the Bayesian ideal embodied in models like the SPRT (see Table 1), simulating these using a consistent set of parameters (detailed in Appendix A). To facilitate understanding of the active ingredients of the model, we also show results from three restricted variants of the full ABS model. The *no-prior variant* removes the adaptive prior (this is equivalent to fixing the prior to Beta(0,0)) while keeping the remaining components. The *direct-sampling variant* uses independent samples instead of the autocorrelated samples while keeping the remaining components. The *fixed-sample-size variant* always uses a fixed number of samples (*N* = 5) to form behaviors for both judgments and behaviors.

### Biases in Probability Judgments

The wide range of systematic biases in probability judgments are perhaps the most direct evidence against purely normative probabilistic models as the basis for a descriptive psychological account. We find that the prior on responses and local nature of the sampling algorithm of the ABS (which help to reduce the computational burden of the model by reusing old and useful computations and using only local knowledge of the posterior distribution respectively) suffice to produce many of these biases. Note that in the ABS, there are no biases in the underlying posterior probabilities; biases arise solely from the algorithmic process by which the posterior is sampled and judgments and decisions are generated.

Using prior knowledge to temper the probability estimates was the basis of the Bayesian Sampler model (Zhu et al., 2020). The ABS works in the same way, except that it uses autocorrelated, rather than independent, samples.^10^ As shown in Figure 3A, the prior in the Bayesian Sampler produces a linear bias toward conservative judgments (Zhu et al., 2020) where people avoid the extremes in their probability judgments (Erev et al., 1994; Fiedler, 1991; Peterson & Beach, 1967). This type of conservatism captures the results of a series of probabilistic identities investigated by Costello, Watts, and colleagues (Costello et al., 2018; Costello & Watts, 2014, 2018), which were constructed by adding and subtracting various mean judgments across combinations of events. While all these identities would equal zero if participants reported coherent probabilities (on average), mean judgments were zero for some identities and substantially different from zero for others. The results from the entire set of identities, including conditional probability judgments of dependent events, were well fit by the Bayesian Sampler’s linear conservatism bias (Zhu et al., 2020). As the average behavior of the ABS is approximated by the Bayesian Sampler especially when the effects of local sampling are not strong (e.g., where there are random initializations of the local sampler), the ABS will produce these results as well.

**Figure 4 fig4:**
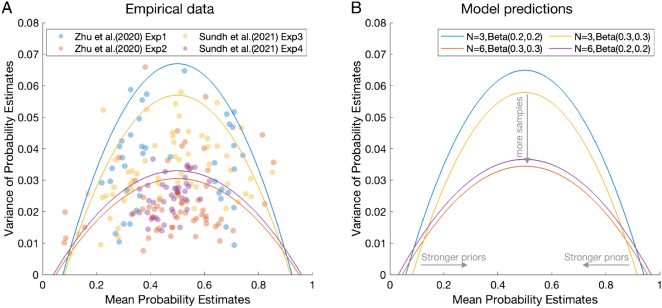
Mean-Variance Relationships in Probability Judgments *Note*. (A) Empirical results based on the four experiments of Zhu et al. (2020) and Sundh et al. (2021), showing inverted U-shaped relationships between means and variances of probability estimates, such that extreme probability estimates (very near 0 or 1) are ruled out. Solid curves are the mixed-effect regression models fitted on individual-level probability estimates. (B) Analytic approximations of the mean-variance relationship predicted by the ABS. The model predicts an inverted-U shape, with stronger priors on responses moving the curve inward and more samples moving the curves downward. ABS = Autocorrelated Bayesian Sampler. See the online article for the color version of this figure.

The sample size and prior on responses of the ABS can be dissociated by examining the mean–variance relationship in probability judgments. When probability judgments are based on sampled outcomes, the relationship between the mean probability estimates and the variance of the estimates will follow an inverse U-shaped (“rainbow-shaped”) curve (see Figure 4A). The prior on responses then constrains the range of possible probability estimates that an agent can produce, thereby lowering the relative variance and pulling the curve both inward and downward (see Figure 4B). For example, for a binary event with a uniform prior, if a single sample is drawn, probability judgments will be either 0.33 or 0.67, and total variance will be relatively lower than for the pure proportions of sampled outcomes (taking now account of the prior on responses). Overall, the Bayesian Sampler predicts a shrinkage of the mean–variance curve for probability judgments, and this was empirically validated in four experiments (Sundh et al., 2021). For the same reasons as the Bayesian Sampler, the ABS model predicts this shrinkage of the mean-variance curve as well (see Figure 4B).[Fig fig4]

Moreover, explicit subadditivity in probability judgments also occurs as a direct consequence of using the prior on responses. Explicit subadditivity is when the estimated probability of an event (*A*_0_) is lower than the sum of estimated probabilities for events (*A*_1_, *A*_2_,…, *A*_*M*′_) where *A*_0_ is the disjunction of those *M*′ mutually exclusive events. That is:

$$\hat{P}(A_{0})<\hat{P}(A_{1})+\hat{P}(A_{2})+\cdots +\hat{P}(A_{M^{\prime }}),$$ 7

where probability theory requires that these should be equal. An explicit subadditivity bias has generally been observed in between-participant designs in which participants were asked explicitly to judge the probability of each of the *M*′ events and their disjunction, *A*_0_, so that a total of *M*′ + 1 probability estimates were recorded (e.g., Tversky & Koehler, 1994). According to the sampling account, for each query, because participants do not know the full range of questions to be asked, they will treat the event to be judged as a binary event; that is, participants will sample instances and noninstances of that event (i.e., *A*_*m*_ vs. not-*A*_*m*_), thus requiring a Beta prior on responses. The resulting estimate of each *P*(*A*_*m*_) will therefore be inflated by regression to the mean. The regression-to-mean effect then applies multiple times on aggregate to the right-hand side of Equation 7 and only once to the left-hand-side, predicting a subadditivity bias for low probability events.

**Figure 5 fig5:**
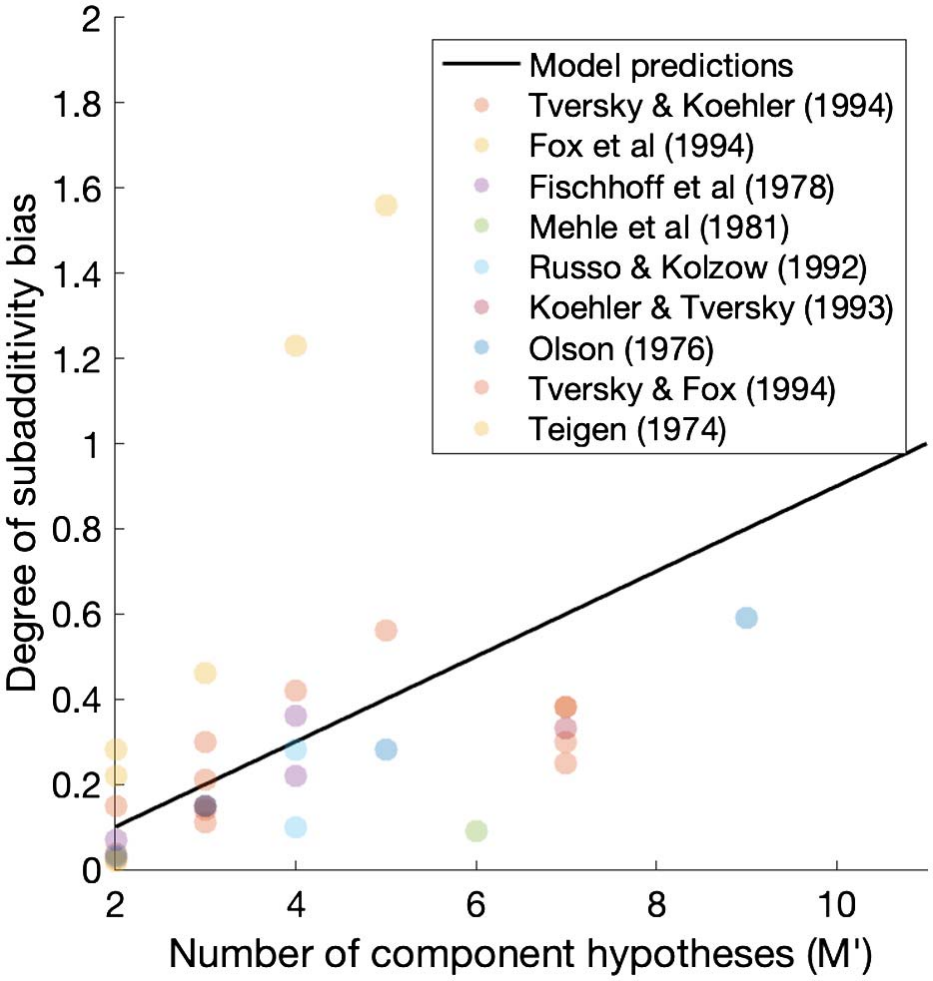
Bias in Explicit Subadditivity, Computed as the Sum of the Probability Estimates of Each Component Hypothesis Minus the Probability Estimates of Their Disjunction, Increases as the Number of Component Hypotheses Increases *Note*. This empirical effect is captured by the Bayesian Sampler (solid line). The sample sizes were set at *N* = 5, and the prior on responses was Beta(1,1). See the online article for the color version of this figure.

As a corollary, the greater the number of disjunctive hypotheses, the more probability judgments will be queried on the right-hand-side of Equation 7, which should lead to a greater degree of subadditivity bias. For *M*′ component hypotheses, the predicted difference between the sum of the *M*′ probability estimates and the probability estimate of the disjunction can be derived as follows:

$$\hat{P}(A_{1})+\hat{P}(A_{2})+\cdots +\hat{P}(A_{M^{\prime }})-\hat{P}(A_{0})=\sum_{m=1}^{M^{\prime }}[\frac{N}{N+2\alpha_{0}}P(A_{m})+\frac{\alpha_{0}}{N+2\alpha_{0}}]-[\frac{N}{N+2\alpha_{0}}P(A_{0})+\frac{\alpha_{0}}{N+2\alpha_{0}}]=(M^{\prime }-1)\frac{\alpha_{0}}{N+2\alpha_{0}},$$

where the assumptions were fixed sample size (*N*) and symmetric prior on responses, Beta(α_0_, α_0_). Indeed, the empirical findings suggest a positive relationship between *M*′ and the degree of explicit subadditivity bias, and the Bayesian Sampler correctly captures the relationship (see Figure 5). Moreover, when the probability of the disjunction of *M*′ mutually exclusive events was exactly 1 (so that one of the disjunctive hypotheses must be true by logic), participants were sometimes asked to only judge the probabilities of *M*′ component hypotheses but not their disjunction. In this case, model predictions can be analytically approximated as $(M^{\prime }-2)\frac{\alpha_{0}}{N+2\alpha_{0}}$ under the same assumption as before. This prediction also matches the empirical pattern known as the binary complementarity: on average, no subadditivity bias was observed for mutually exhaustive events when *M*′ = 2 (Tversky & Koehler, 1994). The ABS model inherits these predictions from the Bayesian Sampler.[Fig fig5]

Similarly, this regression-to-mean effect predicts the conjunction fallacy (Costello & Watts, 2017; Zhu et al., 2020). The conjunction fallacy arises where the estimated probability for a conjunctive event is greater than that for its constituent events $\hat{P}(A_{0}\cap A_{1})\geq \hat{P}(A_{0})$, whereas probability theory requires the probability of conjunctive events to be less or equal with their constituents, *A*_0_ and *A*_1_ (Tversky & Kahneman, 1983). The conjunction fallacy occurs in the Bayesian Sampler when the regression-to-mean applies more to the conjunctive event than to the constituent events. Specifically, it is assumed that fewer samples of the more-complex conjunctive events can be generated or tallied in a fixed amount of time; and the prior thus produces a greater regression-to-mean effect for smaller sizes (see Equation 6). This allows the Bayesian Sampler to predict above-chance levels of conjunction fallacies when the conjunction and constituent event both have low probability (Zhu et al., 2020), as is often the case in experiments (Costello & Watts, 2017). The ABS model also inherits this prediction from the Bayesian Sampler.

In contrast with the explicit judgments of *M*′ + 1 probabilities above, both subadditivity and its opposite effect, superadditivity, have been observed in so-called implicit experimental designs. In implicit designs, only two probability judgments are made: one for the unpacked descriptor (e.g., “baby bottles and other bottles made of glass”) and one for the simple disjunctive descriptor (e.g., “bottles made of glass”; Dasgupta et al., 2017; Sloman et al., 2004). Unpacking to typical examples (e.g., a baby bottle in the category of bottles made of glass) leads to subadditivity: $\hat{P}(A_{0})\leq \hat{P}(A_{1}\cup \;A_{2}\cup \cdots \cup A_{M}),$ whereas unpacking to atypical examples (e.g., a shampoo bottle in the category of bottles made of glass) leads to superadditivity: $\hat{P}(A_{0})\geq \hat{P}(A_{1}\cup \;A_{2}\cup \cdots \cup \;A_{M})$ (Dasgupta et al., 2017; Sloman et al., 2004). Again, since *A*_0_ was unpacked into *M* mutually exclusive events (*A*_0_ = *A*_1_ ∪ *A*_2_ ∪ … ∪ *A*_*M*_), probability theory requires the two probability estimates to be equal. Previous work with autocorrelated sampling models (Dasgupta et al., 2017; Sanborn & Chater, 2016) accounted for this effect by assuming that the descriptor influenced the local sampler’s starting point: typical unpacking initializes the sampler in a high probability region of the hypothesis space, while atypical unpacking initializes it in a low probability region. As a result, the proportion of hypotheses supporting the event’s occurrence will be highest for typical unpacking, intermediate for the simple disjunctive descriptor (assuming it results in a random starting point), and lowest for atypical unpacking. We believe that the ABS will inherit this prediction because it produces autocorrelated samples, though we do not reproduce it here because auxiliary assumptions about the locations and probabilities of hypotheses are needed to do so. This explanation of implicit subadditivity and superadditivity depends on local sampling. They cannot be predicted by the Bayesian Sampler model (Zhu et al., 2020; see a similar argument against a “regressive model” in Tversky & Koehler, 1994), which assumes independent sampling.

Interestingly, people’s probability estimates are also found to exhibit so-called “partition dependence.” That is, they regress to $\frac{1}{M}$ where *M* is the number of alternatives that people are encouraged to consider (see Figure 3B, for a summary; Attneave, 1953; Bardolet et al., 2011; Fox & Rottenstreich, 2003; Varey et al., 1990). For example, asking “what is the probability that Sunday will be hotter than any other day next week?” encouraged participants to treat the event as binary, and their estimates were observed to be biased toward $\frac{1}{2}$, while asking, “what is the probability that the hottest day of the week will be Sunday?,” encouraged participants to consider seven possible outcomes, and estimates were observed to be biased toward $\frac{1}{7}$ (Fox & Rottenstreich, 2003). In ABS, framing the probability query as judging an *M*-variant event invokes a Dirichlet prior with *M* parameters, Dir(α_0_, α_1_,…, α_*M*−1_), which for a binary event reduces to a Beta prior, Beta(α_0_, α_1_). Partition dependence effects can be explained by assuming that people have no a priori reason to believe one event occurs more often than another event: α_0_ = α_1_ = ⋯ = α_*M*−1_ and so probability estimates are predicted to be biased toward $\frac{\alpha_{0}}{\sum_{m=0}^{M-1}\alpha_{k}}=\frac{1}{M}$ (see Figure 3A). In the ABS, the impact of this noninformative prior should be more pronounced in situations where people are less knowledgeable about the probability estimation task or less confident in a learning context (reflecting fewer samples), matching the empirical results (See et al., 2006).

### Choice Accuracy and RT

The Bayesian Monte Carlo process for choice and RT correctly predicts four key relationships between choice and RT. First, and in common with many other evidence accumulation models, the ABS predicts a trade-off between accuracy and speed where increasing decision thresholds lead to, on average, more evidence being accumulated (and thus higher accuracy) as well as longer RT. This trade-off between accuracy and speed has been widely documented in the literature (Garrett, 1922; Johnson, 1939; Pachella, 1974; Ratcliff & Rouder, 1998; Wickelgren, 1977).

Second, unlike many models, the ABS predicts that correct and incorrect responses have unequal average RT. The empirical result is that, when accuracy is emphasized (or in difficult tasks), errors are usually slower than correct responses. By contrast, when speed is emphasized (or in easy tasks), errors are usually faster (Luce, 1986; Ratcliff et al., 2003; Ratcliff & Rouder, 1998; Swensson, 1972). This empirical pattern is surprisingly difficult to match for models that accumulate relative evidence to symmetric bounds: these models predict that the response time distributions for correct responses and errors will always be the same, regardless of choice accuracy (Link & Heath, 1975; Vickers, 1979). To produce slow errors, the usual route is to add variability to the strength of the “signal,” or the drift rate in DDMs (Ratcliff & Rouder, 1998). While both strong signals and weak signals will produce equal mean RT, weak signals are both more error-prone and slower. So, with an equal mixture of strong and weak signals, there will be more slow errors and more fast correct responses.

**Figure 6 fig6:**
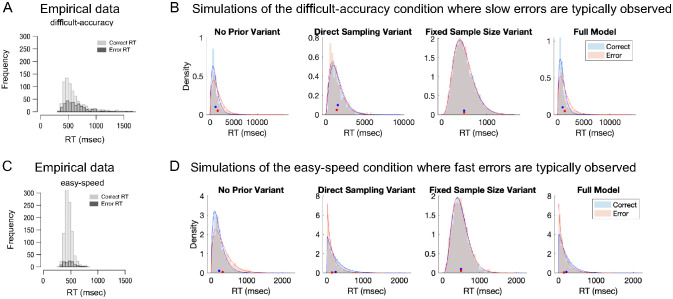
Slow and Fast Errors *Note*. (A) Empirical choice outcomes and response-time distributions in the difficult-accuracy condition. (B) Simulated choice outcomes and RT distributions in the difficult-accuracy condition. RTs were fitted with Gamma distributions with the best-fitting distribution shown as solid lines for correct (in blue) and error (in red) responses. Overlaid dots and their horizontal error bars denote mean RTs and 95% confidence intervals respectively. Similarly, (C) and (D) are respectively empirical data and model simulations for the easy-speed condition. Across the different variants, only the full ABS model reproduces both slow and fast errors in the correct experimental conditions. All predicted RT distributions were unimodal and positively skewed. The full model also correctly reproduces an RT distribution that becomes more positively skewed and spreads out with an increase in the decision threshold. Empirical data were adapted from “Modeling Regularities in Response Time and Accuracy Data With the Diffusion Model,” by R. Ratcliff, P. L. Smith, and G. McKoon, 2015, *Current Directions in Psychological Science*, *24*(6), 458–470 (https://doi.org/10.1177/0963721415596228). Copyright 2015 by Sage Publications. Adapted with permission. ABS = Autocorrelated Bayesian Sampler; RT = response times. See the online article for the color version of this figure.

The ABS produces slow errors in a different way. Instead of adding cross-trial variation to the signal strength, or independent within-trial variation to the signal strength (Diederich & Oswald, 2016), slow errors result from the local sampling algorithm producing autocorrelated samples. For example, if the sampling algorithm begins far above the decision boundary (e.g., the red subspace in the posterior of hypotheses illustrated in Figure 2B), then the initial samples will almost all favor the correct response, while if the sampling algorithm begins far below the decision boundary (e.g., the blue subspace in the posterior of hypotheses) then the proportion of correct samples will almost all favor the incorrect response. Slow errors also require optional stopping, because with a fixed stopping rule the response distribution is itself fixed. This can be seen in the simulation in Figure 6B: both autocorrelation and optional stopping (i.e., the no prior variant) are needed to produce errors that are on-average slower than correct responses.[Fig fig6]

Fast errors, often found in easy tasks, are produced in a different way. The usual route to producing fast errors is to assume variability in the starting point of the evidence accumulation process (Laming, 1968; Ratcliff & Rouder, 1998; Ratcliff et al., 2003). In the ABS, the adaptive prior on responses is assumed to change in response to the outcomes of the preceding trial. This encourages repeating past successes, but also introduces cross-trial variability in the starting point of the accumulator. This is because the accumulator will be biased toward whichever response was correct on the last trial, and assuming that (as is usual in experiments) the correct response randomly varies between trials, it will sometimes be closer to the correct threshold and sometimes closer to the error threshold. For those latter trials, the amount of evidence required to reach the error threshold is reduced, leading to a shortened mean RT for errors. As with slow errors, optional stopping is also necessary: only with both the adaptive prior on responses and optional stopping (i.e., the direct sampling variant) do fast errors appear in the simulation in Figure 6D.

The differences between the simulations of the “difficult-accuracy” condition (Figure 6B) and the “easy-speed” condition (Figure 6D) track the conditions in which slow errors and fast errors are found. We assume that: (a) greater emphasis on accuracy causes the threshold to be higher, and consequentially more pieces of evidence are needed to terminate the sampling algorithms, and (b) easier stimuli makes the evidence more homogenous (e.g., samples are more likely to point to the same response).^11^ As a result, the “easy-speed” condition involves integration over homogenous but smaller amounts of evidence than in the “difficult-accuracy” condition. In other words, the starting point of the accumulator has more influence, while the degree of autocorrelation has less influence, on determining the predicted behavior in the “easy-speed” condition than in the “difficult-accuracy.” Across Figure 6B, D, only the full ABS model matches the empirical observations that slow errors are more common in the “difficult-accuracy” condition, while fast errors are more common in the “easy-speed” condition.

**Figure 7 fig7:**
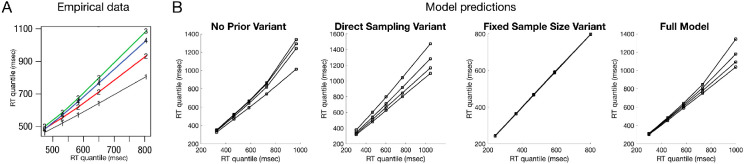
Sample Quantile–Quantile Plots of Response-Times Distributions for Different Levels of Task Difficulty *Note*. (A) An example Q–Q plot of empirical RT distributions adapted from “Modeling Regularities in Response Time and Accuracy Data With the Diffusion Model,” by R. Ratcliff, P. L. Smith, and G. McKoon, 2015, *Current Directions in Psychological Science*, *24*(6), 458–470 (https://doi.org/10.1177/0963721415596228). Copyright 2015 by Sage Publications. Adapted with permission. One difficulty level was selected to compute its quantiles and then quantiles of the other four difficulty levels were plotted against the first condition. The rank of a condition depends on its mean RTs. (B) Q–Q plots of RT distributions produced by the ABS model and its variants. ABS = Autocorrelated Bayesian Sampler; RT = response times. See the online article for the color version of this figure.

As in other models (e.g., Blurton et al., 2020), the assumptions of an exponential waiting time between consecutive samples and the optional stopping rule correctly reproduce many distributional properties of RTs including that (a) there tends to be one mode in the distribution and (b) distributions with higher means are more positively skewed. Further regularities in the shapes of RT distributions were stressed by Ratcliff et al. (2015) using quantile–quantile (Q–Q) plots (see Figure 7A). Plotting the quantiles of RT from one difficulty condition against the quantiles from another difficulty condition, the empirical Q–Q plots reveals near-linear relationships and a fan shape: increasing task difficulty has its greatest impact on the tails of the distribution with the near linearity suggesting similar RT distribution shapes across difficulty conditions. As shown in Figure 7B, the ABS captures the fan shape and near-linear regularity. The direct-sampling variant shows results that are closer to linear, as would be expected if the autocorrelation in samples causes the upper tails in RT distributions to spread out even more in harder tasks. Also of interest is the fixed-sample-size variant, because it always collects the same number of samples for all difficulty levels, produces identical quantiles between RTs from one level of difficulty and those from another, and thus does not match the empirical data.[Fig fig7]

### Confidence in Decisions

From a Bayesian perspective, it is natural to map decision confidence onto the posterior probability that the decision is correct, a mapping which has been called the Bayesian Confidence Hypothesis (Kepecs & Mainen, 2012; Mamassian, 2016; Pouget et al., 2016). For the SPRT, posterior probability is updated as sensory samples are observed, and its posterior probability at the time of choice is simple: it is the posterior probability when the threshold is reached, because as evidence collection stops once this occurs (see Figure 2A confidence). The SPRT thus predicts that decision confidence is determined only by the threshold values, because the threshold captures the amount of evidence favoring one hypothesis or the other. Given that the threshold value is fixed prior to, and independent of, the characteristics of a particular trial, this means that confidence will be the same for all trials on which the same hypothesis is chosen.^12^

Unlike the SPRT, the ABS does not have direct access to the posterior probability that a response is correct (i.e., its confidence). Instead it needs to estimate this probability given a set of samples (see Figure 2B confidence). Fortunately, the form of the adaptive prior on responses (a Beta distribution in the case of binary choice) makes this estimate easy to update as samples are sequentially generated. At the start of the trial, the adaptive prior on responses reflects the prior belief in different probabilities that each response is correct. Using the binary choice example, assume a prior for choice *A* of Beta(*i*, *j*) (and a prior for choice *B* of Beta(*j*, *i*)). When coming to a decision, samples in favor of each response, *S*(*A*) and *S*(*B*), are sequentially collected until the decision process is terminated by the stopping rule. The confidence after *N* samples (i.e., *N* = *S*(*A*) + *S*(*B*)) is then

$${\text{Conf}}_{A}=\frac{S(A)+i}{N+i+j},\;\text{in}\;A\;{\text{Conf}}_{B}=\frac{S(B)+j}{N+i+j},\;\text{in}\;B.$$ 8

The max-minus-next heuristic stopping rule terminates the sampling algorithm when the quantity of evidence favoring one choice exceeds a threshold, Δ=|i+S(A)−(j+S(B))|>0. The final decision confidence can then be rewritten as follows:

$${\text{Conf}}_{A}=1-{\text{Conf}}_{B}=\frac{i+j+N+\Delta }{2(i+j+N)},\;\text{if}\;A\;\text{was chosen},$$ 9

where the confidence judgments predicted by the ABS are decided by both the threshold values (Δ) and the amount of evidence accumulated (*N*; this is the same as the number of samples generated because evidence is directly mapped from hypothesis samples; for example, a sample of 27 dots will be converted into a piece of evidence for the proposition that the number of dots is greater than 25): the greater the number of samples generated before a decision is reached, the lower the confidence in that decision. This is because the ABS embodies a prior over the strength of signal in the Bayesian Monte Carlo process, and the longer the sampling process continues, the more likely the signal is weak, and so that the posterior probability that the decision is correct correspondingly decreases. This is in contrast to the SPRT: in the SPRT, confidence is unaffected by additional sampling because confidence is determined by a fixed decision threshold.

**Figure 8 fig8:**
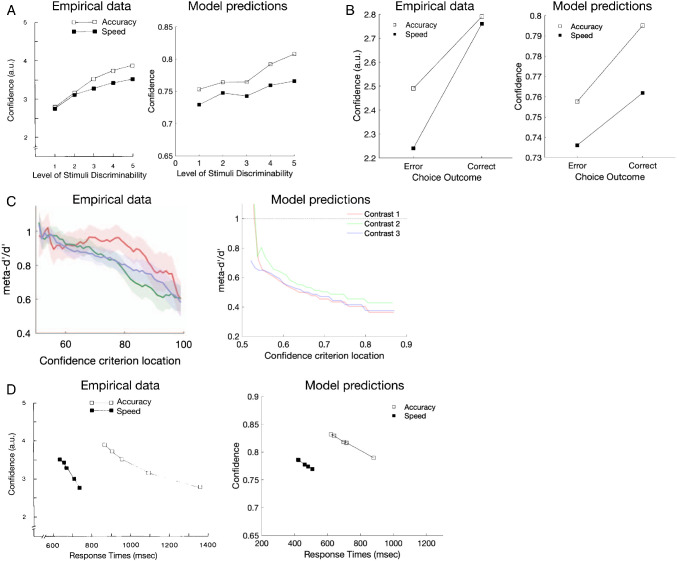
ABS Simulations Showing Effects of Confidence in Decisions *Note*. (A) Positive relationship between stimulus discriminability and average confidence. (B) Resolution of confidence in which average confidence is higher for correct responses than for incorrect responses. Empirical data replotted from Vickers and Packer (1982). (C) Degree of metacognitive efficiency showing extreme confidence ratings are less informative about the accuracy of a choice. (D) Negative (cross-trials) relationship between confidence and RT and positive (cross-conditions) relationship between confidence and RT. Each dot denotes a level of difficulty. Empirical data adapted from Vickers and Packer (1982). Error bars denote 95% confidence intervals of the model simulations. Panels A, B, and D adapted from “Effects of Alternating Set for Speed or Accuracy on Response Time, Accuracy and Confidence in a Unidimensional Discrimination Task,” by D. Vickers and J. Packer, 1982, *Acta Psychologica*, *50*(2), 179–197 (https://doi.org/10.1016/0001-6918(82)90006-3). Copyright 1982 by Elsevier. Adapted with permission. Panel C adapted from “The Nature of Metacognitive Inefficiency in Perceptual Decision Making,” by M. Shekhar and D. Rahnev, 2021a, *Psychological Review*, *128*(1), 45–70 (https://doi.org/10.1037/rev0000249). Copyright 2021 by the American Psychological Association. Adapted with permission. ABS = Autocorrelated Bayesian Sampler. See the online article for the color version of this figure.

The decreasing decision confidence of the ABS with an increasing number of samples allows it to capture four key empirical phenomena which are not accommodated by the SPRT described above: the positive relationship between confidence and the discriminability of the stimuli (Baranski & Petrusic, 1998; Vickers, 1979; Vickers & Packer, 1982, Figure 8A), the “resolution of confidence” effect (Ariely et al., 2000; Baranski & Petrusic, 1998; Garrett, 1922; Vickers, 1979; Vickers & Packer, 1982, Figure 8B), so-called “metacognitive inefficiency” (Shekhar & Rahnev, 2021a, 2021b, Figure 8C), and the complex relationship between RT and confidence (Baranski & Petrusic, 1998; Vickers & Packer, 1982, Figure 8D).[Fig fig8]

The first of these effects, the positive relationship between confidence and stimulus discriminability, follows from variation in the strength of the signal in the ABS. More discriminable stimuli will result in more homogenous evidence supporting one alternative (i.e., samples will more consistently support one response alternative over the other), and because decision confidence is a transformation of the proportion of samples that support the chosen response, more discriminable stimuli will on average produce higher confidence judgments (see Equation 8). Conversely, on more difficult trials, the evidence will be more heterogeneous and so the ABS predicts lower average decision confidence. Figure 8A shows this qualitative effect arising in ABS model simulations, in which confidence is expressed on a probability scale which is ordinally related to the scale with which the empirical data were collected. This pattern is produced by all model variants (see Table 2).[Table tbl2]

Second, average confidence ratings tend to be higher for correct responses than for incorrect responses (e.g., Ariely et al., 2000; Baranski & Petrusic, 1998; Vickers, 1979, 2014; Vickers & Packer, 1982). This so-called “resolution-of-confidence” effect also holds true even if stimulus difficulty is held constant (Baranski & Petrusic, 1998) and even if choice and confidence are simultaneously elicited from participants (Kiani et al., 2014; Ratcliff & Starns, 2009; Van Zandt, 2000). Once again, the SPRT cannot properly explain this effect given that its thresholds are fixed prior to, and independently from, the characteristics of particular trials (e.g., it is constant across all trials or randomly drawn from a fixed distribution). However, if we assume that people have the correct generative model of the task (i.e., the probability of generating a sample that supports the correct alternative is the largest among all other alternatives), the ABS predicts that correct responses will on average be made with higher confidence. This is tied to the explanation for slower errors above: autocorrelations cause errors to be slower on average, and slower responses produce lower confidence judgments (see Equation 9). Therefore, the ABS predicts a resolution-of-confidence effect in experimental conditions that produce slow errors (see Figure 8B). As this effect requires both optional stopping and autocorrelated samples, as also are required to produce slow errors, only the full model and the no prior variant produce it (see Table 2).

Third, studies have shown that the metacognitive judgments in confidence ratings generally carry less information about the accuracy of a decision than would be predicted by a purely normative account like the SPRT. Thus, there seems to be a systematic deficit in “metacognitive efficiency” (Shekhar & Rahnev, 2021a, b). To give an intuition, imagine a participant is asked to make a decision whether to respond *A* or *B* to a stimulus. The participant’s ability to discriminate between the alternatives (i.e., *d*′) can be calculated, based on SDT, by using the percentage of *A* stimuli that are correctly identified (i.e., hits) and the percentage of *B* stimuli that are incorrectly identified as *A* stimuli (i.e., false alarms). This standard *d*′ measure can also be extended to metacognition by choosing a confidence criterion and recalculating the hit and false alarm rates from confidence judgments that exceed this criterion to produce a *meta_d*′.^13^ SDT predicts that *d*′ equals *meta_d′* for any confidence criterion and so predicts that metacognitive judgments are always efficient (while the SPRT predicts constant confidence judgments and so cannot be evaluated using this measure). By contrast, a value of *meta_d′*/*d*′ < 1 would indicate that information available for the decision is lost in part or in whole when making confidence judgments. Empirically, metacognition has been found to be inefficient, and moreover *meta_d*′ decreases relative to *d*′ as the confidence criterion increases, meaning that higher confidence ratings are less informative than lower confidence ratings (Shekhar & Rahnev, 2021a, 2021b). Metacognitive inefficiency has been explained by adding additional noise to confidence judgments (Shekhar & Rahnev, 2021a).

While it would be straightforward to add noise to the ABS confidence judgments, surprisingly this additional noise is not necessary to produce such metacognitive inefficiency; in fact, there are multiple routes for the ABS to produce this effect already offered in the current specification. A first route derives from more informed decisions based on larger numbers of samples being overall less confident decisions. For example, imagine using a stopping rule with Δ = 2 and a symmetric Beta(1,1) prior on responses. If a decision is made based on only a total of two samples, then both will have to be in favor of the chosen response and confidence will be 75% (i.e., plugging these values in Equation 8: $\frac{2+1}{2+2}=75\%$). However, if a decision is made based on a total of 100 samples then only 51 can have supported the chosen alternative (because the stopping rule requires Δ = 51 − 49 = 2) and confidence will be about 51% (i.e., plugging these values in Equation 8: $\frac{51+1}{100+2}\;\approx \;51\%$). Thus, with optional stopping, lower confidence decisions will be based on more samples (and so have longer RTs) and hence will be more informative (see Equation 9). A second route derives from basing confidence judgments on discrete samples of hypothesis counts rather than the Gaussian distributed sensory evidence assumed by SDT; this applies even if samples are independent, a fixed number of samples are generated, and no prior is used (see Appendix D). Therefore, the ABS predicts decreasing metacognitive efficiency for more extreme confidence judgments not only for the full model (see Figure 8C), but also for all its variants (see Table 2).

Finally, confidence is empirically observed to systematically vary with RTs, with positive (cross-condition) and negative (cross-trial) relationships between confidence and RTs (see Figure 8D). When people are forced to respond more quickly in a particular experimental condition, their confidence reduces, which is in line with the standard speed–accuracy trade-off, assuming the confidence positively covaries with accuracy (Irwin et al., 1956; Vickers & Packer, 1982). Both the SPRT and the ABS can capture the positive (cross-condition) relationship simply by varying the threshold according to experimental conditions: emphasizing accuracy moves the threshold further away from the starting point of the accumulator (and vice versa in the speed condition). Higher threshold values in the SPRT lead to more extreme final log odds and therefore higher confidence readouts. Higher threshold values in the ABS (i.e., larger Δ) naturally lead to higher confidence as shown in Equation 9.

However, within a condition, people are *more* confident in decisions they reach quickly—intuitively, the “easy” trials are decided quickly and with high confidence (Baranski & Petrusic, 1998; Vickers & Packer, 1982). As noted above, the SPRT cannot explain this because the strength of evidence at which a decision is made depends only on the threshold, which is determined prior to, and hence independently from, the characteristics of any particular trial. The ABS can explain this negative (cross-trial) relationship because earlier termination (for a fixed threshold Δ) implies that there will be a higher proportion of evidence supporting the chosen alternative. As a result, the ABS predicts that within a condition, faster decisions will be given with higher confidence.

### Confidence Intervals

So far, we have considered confidence in decisions. But confidence reports can also be elicited for estimates by asking for confidence intervals. Commonly a participant is given a probability first and then asked to produce an interval (by giving upper and lower bounds) that correspond to the probability (e.g., “give the smallest interval which you are 60% certain to include the number of dots which appeared onscreen: between ____ and ____ dots”). However, this procedure can also be reversed: participants can be shown an interval of some quantity of interest and then asked to evaluate the probability that the stimulus falls within that interval (e.g., “what is the probability that the number of dots which appeared onscreen falls in the range of 23 to 25?”; Juslin & Persson, 2002).

**Figure 9 fig9:**
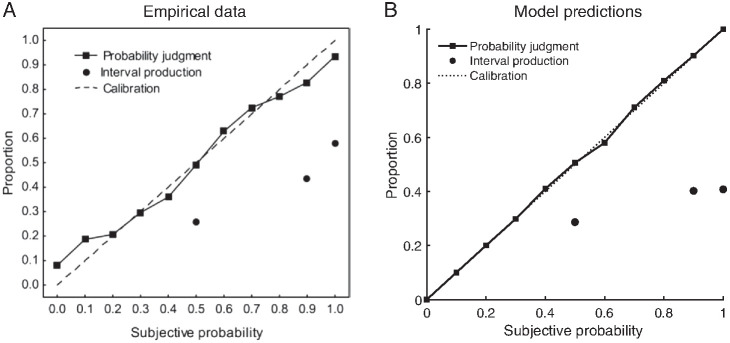
Generating and Evaluating Confidence Intervals *Note*. (A) Empirical data for interval evaluation (i.e., probability judgment) and interval production, adapted from “Calibration, Additivity, and Source Independence of Probability Judgments in General Knowledge and Sensory Discrimination Tasks,” by P. Juslin, A. Winman, and H. Olsson, 2003, *Organizational Behavior and Human Decision Processes*, *92*(1–2), 34–51 (https://doi.org/10.1016/S0749-5978(03)00063-3). Copyright 2003 by Elsevier. Adapted with permission. Interval evaluations were relatively well calibrated while substantial overconfidence was observed in interval production. The dashed line illustrates perfect calibration. (B) ABS predictions of confidence interval production and evaluation: strong overconfidence in interval production (dots) and no overconfidence in interval evaluation (squares). The horizontal axis indicates either the requested interval coverage (production) or the judged probability of the interval (evaluation), while the vertical axis indicates the empirical proportion of events covered by the interval. ABS = Autocorrelated Bayesian Sampler.

In the ABS, confidence interval production and evaluation both are driven by very similar mechanisms to those underlying the naïve intuitive statistician model of Juslin et al. (2007). Taking a set of samples, a confidence interval can be produced by using the lower and upper bounds of the sample coverage (i.e., empirical quantiles of the samples) as the lower and upper bounds of the confidence interval. When the values of the quantiles are not explicitly represented in the sample (e.g., deriving a 93% CI based on 5 samples), linear interpolation was assumed to fill in the gap between the samples (Juslin et al., 2007). This mechanism correctly predicts the considerable overconfidence in interval production found empirically (see Figure 9A, dots; Juslin et al., 2003, 2007). This is because for small sample sizes, the empirical quantile of the sample will have a shorter range than the confidence interval from the posterior because distributional tails tend to be underrepresented within a few samples. Therefore, the proportion generated from the sample will be too small, producing an overconfident interval in our simulations (see Figure 9B, dots). One might then question why interval production overconfidence is not corrected in the same manner described for probability judgments above where useful prior knowledge is incorporated—this lack of correction for confidence interval production is what was “naïve” about the naïve intuitive statistician model. Corrections for intervals, however, depend on the functional form of the distribution, so that a general correction process is difficult to establish in the ABS. While the standard computation of a confidence interval assumes a Gaussian distribution, for unknown distributions confidence intervals are usually produced by bootstrapping. For the purposes of producing the confidence interval for a sample, as opposed to producing the confidence interval for a mean, bootstrapping is essentially what the ABS does.[Fig fig9]

In contrast to confidence interval production, confidence interval evaluation shows very different empirical results: here there is little to no overconfidence with only a small degree of conservatism at the extremes of subjective probability (see Figure 9A, squares; Juslin et al., 2003). This arises in the ABS (see Figure 9B, squares), using the simplest possible assumption (and following Juslin et al., 2007) that people answer this question by generating samples and calculating the proportion that fall within the provided interval. As noted by Juslin et al. (2007), this proportion is an unbiased estimator, and hence shows good calibration.^14^

### Decisions Affecting Later Estimates

Besides eliciting confidence judgments after choices, experimenters have often asked participants to provide separate secondary responses to the same stimulus. One example is the decision–estimation task where people first choose, say, whether the number of dots which appeared on-screen was greater or smaller than 25, and then are asked, immediately following the choice, to estimate the number of dots. In this setting, an estimate can be influenced by the preceding choice (e.g., Jazayeri & Movshon, 2007; Tversky & Kahneman, 1974). In cognitive judgments, estimates have often been observed to be pulled toward a preceding arbitrary value—the well-known phenomenon as the *anchoring* bias (Epley & Gilovich, 2006; Tversky & Kahneman, 1974). For example, in the famous study of Tversky and Kahneman (1974), participants were first asked to choose whether the percentage of African countries in the United Nations was higher or lower than a value (h*), and then give an estimate of that percentage. The comparison value used in the choice, h*, was seen to be generated randomly and so should have been irrelevant to the distribution of hypotheses (and thus irrelevant to the estimate too), but estimates were biased toward h*.

However, in an almost identical paradigm of decision–estimation tasks, perceptual judgments showed the opposite effect: estimates of aspects such as dot orientation or direction of motion have been observed to be pushed away from h* (Jazayeri & Movshon, 2007; Luu & Stocker, 2018; Zamboni et al., 2016). The phenomenon is better known as the *repulsion* effect found in perceptual tasks.

Existing models of anchoring cannot predict the repulsion effect and vice versa. This is because they only predict one direction of bias (e.g., Jazayeri & Movshon, 2007; Luu & Stocker, 2018; Strack & Mussweiler, 1997; Tversky & Kahneman, 1974), and thus fail to capture the co-occurrence of anchoring and repulsion. While this would be tenable if anchoring and repulsion were each specific to their respective (cognitive or perceptual) domains, a recent empirical investigation suggests otherwise (Spicer et al., 2022a). In this work, we noted that the location of the comparison value, h*, relative to the distribution of hypotheses has not typically been the same across cognitive and perceptual paradigms. Indeed, it was found empirically that the relative location of h* determines whether the subsequent estimates will be pulled toward or pushed away in both cognitive and perceptual tasks. Specifically, estimates of the stimulus value are drawn toward values of h* which are distant from the true value of the stimulus (replicating the anchoring effect) but pushed away from values of h* which are near to this true value (replicating the repulsion effect; Spicer et al., 2022a). This finding is consistent with a common general-purpose algorithm underlying decision-making in both cognition and perception.

**Figure 10 fig10:**
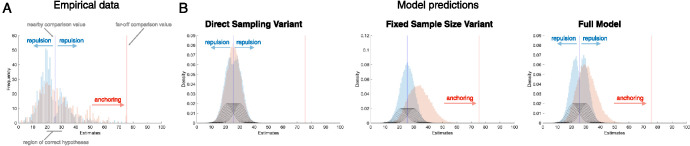
Anchoring and Repulsion Effects *Note*. (A) Experimental data on the decision-estimation task. For the region of correct hypotheses in the range of [21, 30], the estimates were pushed away from a nearby comparison value (25.5) used in the preceding decision task (blue bars), while pulled toward a far-off comparison value (75.5; red bars). The data was replotted from Spicer et al. (2022a). (B) Simulating the ABS (right), its direct sampling variant (left), and fixed sample size variant (middle) on decision-estimation tasks. The full ABS model predicts both anchoring (for far-away target stimuli; red bars) and repulsion effects (for close-by target stimuli; blue bars), whereas the direct-sampling variant only predicts the repulsion effect and the fixed-sample-size variant only predicts the anchoring bias. Target Gaussian distributions for sampling with means in the range of [21, 30] were shown in black solid lines, which were vertically rescaled by a factor of 1/4 to aid visualization. ABS = Autocorrelated Bayesian Sampler. See the online article for the color version of this figure.

The anchoring effect, the repulsion effect, and their dependence on the relative position of h* are captured by the ABS assuming that the set of samples generated to make the choice is then reused to produce the estimate rather than expending further cognitive resources on generating new samples, thus creating a link between these responses. Each effect is then attributable to one of the core components of the model when making the initial choice: anchoring is produced by the autocorrelated sampling algorithm, and repulsion by the optional stopping rule. To explain anchoring, the ABS follows the approach of Lieder et al. (2018) and assumes that local sampling algorithm uses h* as an initial hypothesis. For a small number of iterations, the local sampler will then be biased toward the initial hypothesis. Anchoring is then produced in our simulations for the full model and all variants except the direct sampling variant (see Figure 10 and Table 2).[Fig fig10]

To explain repulsion, we first note that in the ABS h* effectively partitions the hypothesis space into two binary response regions. The sampling algorithm is adaptively terminated when a sufficient number of samples support one alternative over the other, with the amount determined by the threshold parameter (i.e., Δ). This adaptive stopping rule produces a repulsion bias if the estimate is also based on the same set of samples (Zhu et al., 2019), because the sampling process will terminate only when the weight of evidence favors one hypothesis rather than when the evidence is finely balanced: In effect, optional stopping for choice biases the subsequent estimate away from indifference (i.e., the decision boundary). Thus, repulsion is produced by the full model and all of the variants except for the fixed sample size variant (see Figure 10 and Table 2), and a larger sample size for the initial decision would reduce both anchoring and repulsion effects.

### Cross-Trial Autocorrelations in Estimates and RTs

Substantial cross-trial autocorrelations are an important, and often unexplained, aspect of human behavior. For example, long-range dependencies in estimates and in RTs known as 1/*f* noise have been observed in many cognitive and perceptual tasks and can explain more variance in behavior than the experimental manipulations (Gilden, 2001; Gilden et al., 1995; Wagenmakers et al., 2004).^15^ In these tasks, participants were instructed to repeatedly estimate fixed physical quantities (e.g., a 1-s temporal interval or a 1-in. spatial interval) or to repeatedly choose between two options. The statistical features of the time series produced by participants were analyzed in the frequency domain, with the high-frequency components corresponding to trials that are close together, whereas low-frequency components correspond to trials that are well separated. The power of each of these components for explaining the time series are then calculated (using a spectral density analysis; Gilden, 2001; Gilden et al., 1995; Sheu & Ratcliff, 1995). Standard statistical processes show different relationships between frequency and power: in a random walk power falls off with 1/*f*^2^ noise (i.e., a slope of −2 in log-log power spectra), whereas white noise (also called independent sampling or direct sampling) has a flat power spectrum (i.e., 1/*f*^0^ noise and a slope of 0). In a time-series containing long-range serial dependence, as is typical in human data, power spectra typically have a slope between −1.5 and −0.5, and are thus categorized as 1/*f* noise. The long-range autocorrelations in 1/*f* noise are not straightforward to produce, generally requiring complex processes to do so (Gardner, 1978).

**Figure 11 fig11:**
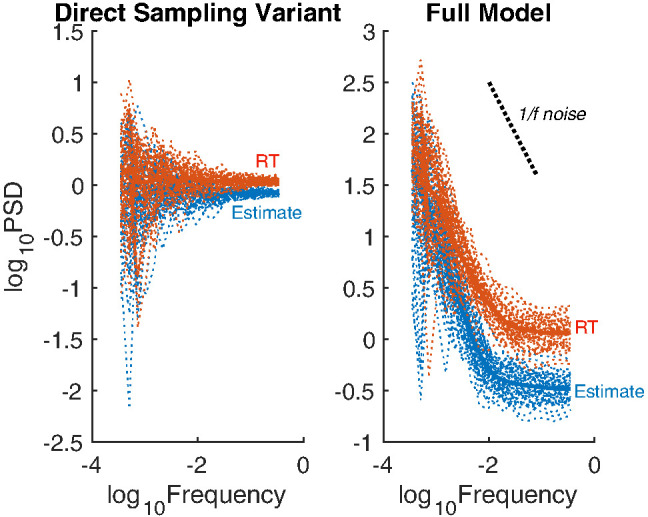
Power Spectra for Time Series of Estimates and RT *Note*. Dashed lines denote power spectra by a simulated participant with solid lines showing the average. RT time series are colored in red, whereas time series of estimates are in blue. (Left) The direct sampling variant predicts independent estimates and RTs and thus exhibits a flat line (i.e., the power spectrum of white noise). (Right) The ABS model predicts autocorrelations in estimates and RTs with the latter displaying flatter slopes than the former (i.e., the power spectra of 1/*f* noise). ABS = Autocorrelated Bayesian Sampler; RT = response times. See the online article for the color version of this figure.

Further complicating the picture, while time-series of estimates have long-range autocorrelations that are classed as 1/*f* noise (Gilden, 2001; Gilden et al., 1995; Wagenmakers et al., 2004; Zhu et al., 2021), RT time series fluctuate as 1/*f* noise but with a log-log slope that is shallower than that of estimates (Van Orden et al., 2003; Wagenmakers et al., 2004). As shown in Figure 11, the ABS qualitatively reproduces the observed autocorrelations in time series of RTs and estimates. The cross-trial autocorrelation in estimates is predicted by the cross-trial carryover of the sampler’s location in the autocorrelated MC^3^ algorithm (Zhu et al., 2018; Zhu, León-Villagrá, et al., 2022): the initial location of the sampler for the present trial is the last sample for the preceding trial. In comparison, the RT time series is predicted to be less autocorrelated because samples generated by the MC^3^ are accumulated to a threshold to produce the RT; this is a nonlinear transformation of autocorrelated samples which “whitens” the power spectrum. Simulations of the full model demonstrate both these effects, and as the effects are driven by the MC^3^ algorithm it occurs for all variants except for the direct sampling variant (see Figure 11 and Table 2).[Fig fig11]

### Summary

Using a consistent set of parameter values, we have shown that the ABS qualitatively captures empirical results and relationships observed across probability judgments, estimates, confidence intervals, choices, confidence judgments, and RT (see Table 2). The wide range of predicted behaviors is based on an internal probabilistic model using a fine-grained set of hypotheses. The process of inferring the posterior probability of the hypotheses is governed by Bayes’ rule and approximated using an autocorrelated sampling algorithm. While the autocorrelation in the sampling algorithm is motivated to make the sampling process computationally efficient, it turns out to be crucial for explaining many empirical effects such as slow errors, anchoring, and cross-trial autocorrelations. Assuming each sample generated is costly, turning these samples into choices relies on an optional stopping rule that trades the benefits of larger samples against the cost of sampling. In turn, the optional stopping rule helps explain empirical effects such as the repulsion effect, the resolution of confidence, and metacognitive inefficiency. The probabilistic model also learns from trial history, using the adaptive prior. This prior helps explain effects such as conservatism, the conjunction fallacy, partition dependence, and fast errors. As shown in Table 2, all components are necessary to explain the full range of behavior. The ability of this model to account for such a wide assortment of human behavior, as we discuss further below, is evidence for this rational process: that people generate samples from a probabilistic representation and then make simple and sensible use of the samples to produce behavior.

## Comparison With Competing Models

There are many models that can produce at least a subset of the empirical effects that the ABS does, and many were briefly mentioned in the text above. Here we compare the ABS first to other models of probability judgments and then to drift-diffusion models of choice, response time, and confidence.

### Models of Probability Judgments

Intensive modeling efforts have also been directed at explaining human probabilistic judgments, spurred on by the identification of biases, particularly those summarized in Table 1, demonstrating that people’s judgments systematically deviate from the rules of probability theory (e.g., Costello & Watts, 2014; Dasgupta et al., 2017; Hilbert, 2012; Nilsson et al., 2009; Peterson & Beach, 1967; Tversky & Koehler, 1994; Zhu et al., 2020). Many models have assumed that probability judgments follow a deterministic process, albeit one that violates the rules of probability theory. For example, one type of model, geared toward accounting for conjunction fallacies, assumes that probability estimates of conjunctions are the weighted average of the probabilities of their constituent events, which produces above-chance conjunction fallacy rates and can reproduce several probabilistic identities (Fantino et al., 1997; Nilsson et al., 2009, 2016). However, these models require additional mechanisms to match the empirically observed combination of above-chance and below-chance rates of conjunction fallacies that the ABS can produce (Fisk & Pidgeon, 1996; Nilsson et al., 2009).

A different type of deterministic approach, at least in the way it has been implemented to explain conjunction fallacies, is based on quantum probability. Here probabilities are based on projections of event subspaces. If the events are compatible, probability judgments are indistinguishable from classical probability theory, but if the events are incompatible then interference produces probability judgments that deviate from classical probability theory. These deviations are such that conjunction and disjunction fallacies will occur at rates above chance, and in this way, quantum probability can produce both above-chance and below-chance conjunction fallacies depending on how the events are represented (Busemeyer et al., 2011). Quantum probability has explained a wide range of probabilistic biases, including some not covered here (Pothos & Busemeyer, 2022). However, there are also probabilistic identities that quantum probability cannot reproduce, that are predicted by sampling-based models, including the ABS (Costello & Watts, 2018; Zhu et al., 2020).

A third deterministic approach is support theory, which was developed to explain subadditivity biases. The core assumption of support theory is that the probability of event descriptions is evaluated rather than the probability of the events themselves and does not incorporate the probabilities of events that are not immediately available (e.g., those not mentioned in the descriptor of events). This approach elegantly explains both a range of implicit subadditivity results, as well as explaining why subadditivity does not occur for mutually exclusive binary events. However, it does not produce the later finding that an atypical unpacking of events produces implicit superadditivity (Sloman et al., 2004), and requires additional mechanism such as an “ignorance prior” which pulls probability judgments toward indifference between the available responses (Fox & Rottenstreich, 2003). These different mechanisms have echoes in the ABS. In the ABS, a hypothesis is “available” only if it has been sampled, and the event description influences the starting point of the local sampler. Further, the prior on responses is a principled version of the ignorance prior, one that is uncertain about the underlying probabilities because often only a small number of samples is available.

Recent approaches have rejected purely deterministic approaches and explored the alternative possibility that stochastic mechanisms explain the biases in probability judgments. For example, simple unbiased response noise has been shown to produce subadditivity (Bearden et al., 2007; Brenner, 2003). However, unbiased response noise alone does not explain why subadditivity still occurs for median judgments. A more promising alternative is to consider corruptive noise in memory or evidence accumulation, which can produce stronger biases (e.g., Costello & Watts, 2014; Erev et al., 1994; Hilbert, 2012). In a leading stochastic model, Probability Theory plus Noise (PT+N), people are assumed to first draw independent samples from a probabilistic representation, and unbiased “counting noise” is added to individual samples to reflect an error-prone cognitive system (Costello & Watts, 2014). This counting noise pulls probability judgments toward indifference and allows the PT+N to capture empirical results such as explicit subadditivity, the conjunction fallacy, and a wide range of probabilistic identities (Costello et al., 2018; Costello & Watts, 2014, 2017, 2018). The PT+N has impressive empirical coverages, although it has recently been argued that it does not fully reproduce all the mean-variance relationship in probability judgments (Sundh et al., 2021): while it will produce the inverted U-shaped relationship between the mean and variance of judgments, the curve will not be pulled inward eliminating extreme (near 0 or 1) probability judgments as is observed in the empirical data and as the ABS predicts. This mean–variance relationship also stands as a challenge to deterministic models because it is not easily produced by simply adding response noise to a deterministic model.

### Drift-Diffusion Models

One important family of models that deserves more extensive discussion is the family of DDMs (Drugowitsch et al., 2012; Gold & Shadlen, 2007; Krajbich & Rangel, 2011; Ratcliff, 1978; Ratcliff et al., 2016). While there are many members of this family, they all describe decision-making as a stochastic process similar to that of a biased random walk (or a biased diffusion process, in continuous time) in which the path of the accumulator is, on average, biased by the drift rate (Bogacz et al., 2006; Ratcliff & McKoon, 2008). For binary choices, this is determined by the difference in the evidence signals supporting the two alternatives. In line with the stopping rule of the SPRT, the threshold reached in the DDM decides the choice and the time taken to do so the response time. However, unlike the static summation process of log-likelihood ratios in the SPRT, the accumulator in the DDM also diffuses because the accumulator is corrupted by noise (typically white noise). In perceptual tasks, the drift rate is related to which choice is objectively correct (Ratcliff & McKoon, 2008), whereas in high-level cognitive tasks where people are choosing their preferred option the drift rate is assumed to be related to the relative appeal of the alternatives (Krajbich et al., 2010; Krajbich & Rangel, 2011).

There are generally strong theoretical links between these models and the normative framework of the SPRT: in the continuous limit, the SPRT converges on the DDM and the drift rate and the corruptive noise in the DDM can jointly mimic the calculation of likelihood ratios in the SPRT (Bogacz et al., 2006). However, implementing an optimal statistical decision test in the form of the DDM also generates a number of useful theoretical and empirical insights that were not originally part of the SPRT. First, the psychologically implausible assumption that people are required to have global knowledge of the generative model of the task to calculate the exact likelihoods (e.g., $P(s_{t}|A)$ or $P(s_{t}|B)$) is implicitly relaxed by the DDM because the drift rate and diffusion noise are free parameters that are recovered from fitting to behavioral data. Thus, the DDM does not need to calculate with the exact cumulative differences in evidence as supposed by the SPRT, greatly improving the DDM’s computational plausibility, given that the exact likelihood ratios are almost always impractical to compute in real time except in simple toy problems.

Second, extensions of the DDM can also account for empirical features such as those noted above which are not accounted for by the SPRT. For example, slow and fast errors (Ratcliff & Rouder, 1998; Townsend & Ashby, 1983) can be produced by further assuming that model parameters are variable across trials (Laming, 1968; Ratcliff, 1981; Ratcliff & Rouder, 1998; Rouder, 1996). In particular, varying the drift rates trial-by-trial generates slow errors, while varying the starting point of the accumulator predicts fast errors (Laming, 1968; Ratcliff & Rouder, 1998). The ABS model works in a similar fashion, as autocorrelation acts like variability in drift rates and a biased prior of evidence acts like variability in the starting point of the accumulator.

Moreover, the benefits of using the DDM instead of the SPRT also apply to explaining confidence judgments. The SPRT predicts the confidence ratings to be identical between correct and incorrect responses because, for a fixed symmetric threshold, there will always be the same level of evidence difference accumulated favoring the selected option,^16^ and the probability that the chosen option is correct is simply read out from the final state of the accumulator. As outlined above, such predictions are contradicted by empirical data in which choice accuracy and confidence are positively related (Baranski & Petrusic, 1998; Dougherty, 2001; Vickers, 1979; Yeung & Summerfield, 2014). To reconcile the confidence data with the SPRT, one kind of DDM introduced another assumption in which the same drift-diffusion process continues to run for a period of times after the choice has been made but before the confidence judgments (Pleskac & Busemeyer, 2010). As the accumulator has a bias toward the correct choice, this continued accumulation after the choice and before the confidence judgment, no longer bounded by the fixed threshold of the decision, will drive the confidence ratings toward supporting the correct choice. Hence, with this additional assumption, this DDM can correctly predict that people should report higher confidence in correct responses than in errors, and moreover that the resolution of confidence effect grows with the delay between choosing and reporting confidence. However, since the temporal structure supposed by this assumption is that confidence occurs after the choice, this DDM cannot explain why the relationship between choice accuracy and confidence also appears when confidence judgments are given simultaneously with a decision (e.g., Kiani et al., 2014; Li & Ma, 2020). In explaining these data, researchers have assumed that confidence decreases with response time (Calder-Travis et al., 2020; Kiani et al., 2014), and as is predicted by the ABS.

Alternative versions of the DDM, such as the RTCON model, have been developed to capture no-choice confidence rating (Ratcliff & Starns, 2009). RTCON assumes that each confidence rating has an independent diffusion process and the first diffusion process to reach the threshold determines the confidence rating. So, for seven confidence ratings, there are seven diffusion processes racing to the threshold. RTCON captures many key empirical relationships between confidence and RT (Ratcliff & Starns, 2009), but appears to have difficulty capturing the interaction between confidence and choice when these judgments are made sequentially (Pleskac & Busemeyer, 2010). The approach of RTCON has been generalized to continuous-response paradigms in which there are an infinite number of potential responses: the circular diffusion model (Smith, 2016) and the spatially continuous diffusion model (Ratcliff, 2018). The two models have recently been integrated into a unified framework where geometric similarity among response options is represented (Kvam & Turner, 2021).

Overall, the family of models encapsulated by the DDM successfully describe behavior in tasks far different from the perceptual tasks for which it was initially developed (Bogacz et al., 2006; Ratcliff et al., 2016), including tasks in which there is little to no perceptual noise, such as value-based decisions (Busemeyer & Townsend, 1993; Milosavljevic et al., 2010; Usher & McClelland, 2004) and recognition memory tasks (Ratcliff et al., 2004, 2011; Starns & Ratcliff, 2014). Because of its strong normative underpinnings in the SPRT, and its psychologically plausible assumptions, the DDM has been widely used in psychology, economics, and neuroscience (Drugowitsch et al., 2012; Fehr & Rangel, 2011; Ratcliff & Smith, 2015; Ratcliff et al., 2016). Indeed, the DDM has become the default framework in many areas of decision-making research. That being said, the broader scope of behavioral responses including choice, RT, confidence, and estimates captured by the ABS have not yet been united within a single implementation of the DDM. This is partly because the task representation needed for choice is different than that needed for estimates or confidence intervals (crucially, the DDM represents an accumulated value—a summary statistics of the sample—but does not retain the sample itself). Thus, while the ABS and the DDM share similar descriptive capabilities, the ABS arguably has the advantage in terms of parsimony given the breadth of behavior covered within its single framework, capturing relationships, such as the combination of anchoring and repulsion effects described above, not explained by current DDM approaches.

## Toward Complete Task-Specific Models

The ABS so far has explained the generic judgment and decision-making process. But how this might be applied to specific tasks? Fortunately, we can relate our approach to existing models in the literature that take quite a similar approach to that taken here, and indeed have helped inspire our work. Here we describe two successful existing models, Nosofsky and Palmeri’s (1997) exemplar-based random-walk (EBRW) model and Blurton et al. (2020) visual attention model, and we outline how using the ABS would involve only minor modifications (such as adding autocorrelations) to them. Thus, we can view these existing models as complete task-specific models in the ABS framework.

The EBRW operates in a hypothesis space of exemplars which represent categories (Nosofsky & Palmeri, 1997). This representational assumption is inherited from the *generalized context model* where each individual exemplar is situated as a point in a multidimensional psychological space, and similarity between exemplars decreases with the distance between points in the space (Nosofsky, 1984; Shepard, 1987). Building on this representation, EBRW further assumes that exemplars are retrieved sequentially as in a random walk process, predicting the time course of categorization and recognition decision-making (Nosofsky & Palmeri, 1997). Similar ideas can be found in the PRW model of visual attention (Blurton et al., 2020; Bundesen, 1990). In this model, a series of tentative categories (i.e., hypotheses) is proposed and accumulated until one category has accrued enough samples more than any other category (Blurton et al., 2020). The generation of tentative categories is governed by the theory of visual attention (Bundesen, 1990).

Across the two models, there is a common mechanism that integrates over hypotheses for response selection. While neither model was originally motivated from normative principles, they can both be seen as special cases within our framework in which decisions are driven by samples of hypotheses (see Appendix B, for detailed comparisons), but with the addition of features such as autocorrelated samples. More specifically, the exemplar-based representation of hypotheses of the EBRW can be adopted by the ABS when modeling categorization tasks, suggesting how the ABS could be applied to explain the effects of similarity and practice in categorization and RT, which have been captured by the EBRW. When combined with a Bayesian theory of visual attention, the ABS could also be generalized to account for human eye movement and object localization. In addition, the correspondence between the PRW and the direct sampling variant of the ABS provides a bridging condition that allows the ABS to account for detailed fits in response time distributions. Furthermore, adding autocorrelations and an adaptive prior to both the EBRW and PRW models generalizes these models to capture a wider range of empirical effects such as those found in confidence judgments. This demonstrates how the ABS can be applied to, and work well in, specific tasks.

## Discussion

The ABS is a step toward a unified rational process of human behavior. Through our analysis, we have identified two key ideas that are necessary for such a unified framework: probabilistic models and approximate inference. Approximate inference via accumulating hypothesis samples means that the ABS views response time in a different way from most existing approaches, as primarily determined by the time required to mentally sample hypotheses, rather than to accumulate more sensory data. We discuss this further below, pointing to possible reconciliations regarding these views of the role of time as well as possible reconciliations between diffusion processes (and noisy probability judgment models) and the ABS. Next, we explore how the ABS could be extended both to multialternative tasks and how complex probabilistic representations could be incorporated. Finally, we discuss and comment on the extent to which the ABS has a rational, Bayesian, basis, and the prospect of quantitatively fitting the model to psychological data.

### Contrasting Views on the Role of Time

The existing dominant view on the role of time in decision-making is to collect and integrate sensory evidence. In the binary choice example, the likelihood ratio between the two alternatives is represented exactly at any moment and the odds of the correct response are constantly updated in light of new sensory evidence or newly retrieved memories. This view has been adopted by many models including SDT, the SPRT, and by and large DDM approaches.

By contrast, the ABS takes a very different view on the role of time because it sees perception and cognition as emerging from probabilistic representations and computations. Instead of coding a single value of the sensory input, people are assumed to implicitly encode multiple values of the sensory input with their subjective uncertainty about those values. But this posterior is difficult to represent exactly for virtually all cognitive tasks; thus, approximation is needed to access it. The passage of time is then viewed as being used for generating more samples from the posterior to refine this approximation. Thus, time matters because it allows the computational process of sampling the posterior to unfold, not because additional sensory data must be accumulated. In the limit, the gradual refinement of the posterior belief should lead to a convergence to the optimal choice.

These contrasting perspectives were also studied in more detail in Lengyel et al. (2015) which presents empirical evidence supporting the view of posterior approximation. It is, however, important to note that the two views on the role of time are not mutually exclusive. One possible reconciliation could be that the brain first conducts evidence integration, and then a posterior based on the sensory evidence can be approximated with sampling, or these processes could be overlapping. Further analyzing the aspects of the two views on the role of time may be an important topic for future research.

### Diffusion, Noise, and Sampling

While we have contrasted other models and the ABS, there are also potential links between these approaches. Considering stochastic models of probability judgments, it may be possible to extend a sampling model with noisy counting, such as the PT+N, to explain the vast majority of the effects that we explored above. In our previous work, we have shown that the PT+N and the Bayesian Sampler models can mimic one another’s predictions of average probability judgments (Zhu et al., 2020). Building on this, it is possible to envision generalizing the PT+N in the same way we generalized the Bayesian Sampler to the ABS. First, rather than using independent samples, the PT+N could instead use a local, autocorrelated sampler such as MCMC or MC^3^. Indeed, Costello and Watts (2018) have begun to explore this possibility by introducing a type of autocorrelation or carry-over effect allowing earlier samples to influence later probability judgments. Second, when making decisions, PT+N could also use optional stopping rather than a fixed number of samples to account for RT data. This could be a promising alternative to the ABS, although more work is required to develop this rough sketch into a formal model and determine how well it reproduces human data.

For diffusion models, an interesting starting point is the recent interest in continuous diffusion models (Kvam, 2019; Kvam & Busemeyer, 2020; Kvam et al., 2022; Ratcliff, 2018). The main focus of the continuous diffusion models was to describe the cognitive processes underlying tasks that involve continuous responses such as orientation estimation (Ratcliff, 2018) and pricing (Kvam & Busemeyer, 2020). The accumulator is typically depicted in a two-dimensional space. Without any bias in starting point, the accumulator initializes in state [0, 0] (i.e., the origin). The amount of evidence accumulated is described as the distance from the origin, and there is a directional bias toward the option that is most favored at that moment. A continuous absorbing threshold (e.g., a semicircle whose center is the origin) defines the space of possible trajectories; when the threshold is reached, the diffusion process is terminated and a response is triggered. The size of the threshold regions that correspond to each response has been adjusted to make some responses more or less likely (Kvam & Turner, 2021).

This kind of mechanism could link a continuous diffusion process to the ABS. As long as the size of the threshold associated with each discrete fine-grained hypothesis is proportional to its posterior probability, then the continuous diffusion process will effectively be sampling from the posterior on fine-grained hypotheses.^17^ If the fine-grained hypotheses that are sampled are then processed to produce judgements and decisions as in the ABS, this would formally link the models, through substituting a diffusion-based sampling algorithm for the local sampling algorithm currently used. Thus, the distinction between diffusion and sampling may not be clear cut, and they may potentially be viewed as parts of a larger framework.

### Multialternative ABS

Asking people to choose among more than two alternatives is often a useful strategy to test the generalizability of computational models developed to explain binary choice data. As previously noted, many aspects of the ABS are readily applicable to such choices: the hypothesis space can be divided into as many regions as required by the query, and the Beta prior generalizes to a Dirichlet distribution when considering more than two options. The optimal stopping rule derived from dynamic programming is, however, more difficult to calculate within realistic times when choosing between more than two alternatives (see Appendix C, for detail). As we have noted, the max-minus-next heuristic is often considered as a good approximation to the optimal stopping rule in multialternative choices (Dragalin et al., 1999, 2000) and, indeed, there is computational work suggesting that humans adopt the max-minus-next stopping rule to choose among many alternatives (Brown et al., 2009). Indeed, recent work in evidence accumulation models find that a variety of binary and multiple-choice phenomena can be modeled as accumulating “advantage”—the difference in evidence supporting one versus another, which is conceptually related to the max-minus-next stopping rule (Miletić et al., 2021; van Ravenzwaaij et al., 2020), and the ABS could be equivalently implemented in this framework. This heuristic stopping rule can also explain the best-known empirical result on the relationship between choice and RT, Hick’s Law (Brown et al., 2009): that RT increases logarithmically with the number of alternatives (Hick, 1952; Proctor & Schneider, 2018).

Multialternative choice tasks with confidence have also recently been used to argue against the Bayesian confidence hypothesis—that confidence in a choice is the posterior probability of that choice. Li and Ma (2020) found that the best-fitting model for confidence ratings in a three-alternative choice task was *not* the posterior probability of the chosen option, but the difference between the probability of the chosen option and the probability of second most probable option. This result could be reconciled with the Bayesian confidence hypothesis through the ABS, assuming a stopping rule such as the max-minus-next heuristic. More formally, consider a three-alternative choice with options *A*, *B*, and *C*, with respective accumulated evidence *i*, *j*, and *k*. Further assuming that *i* > *j* > *k*, then option *A* is chosen where *i*−*j* = Δ following the max-minus-next stopping rule. The confidence difference between the best and the second-best is thus, $\text{Diff}={\text{Conf}}_{A}-{\text{Conf}}_{B}=\frac{i-j}{i+j+k}$, and the total amount of evidence is related to this difference, $i+j+k=\frac{i-j}{\text{Diff}}$. Given that, we can rewrite the confidence for the chosen option *A* as follows:

$${\text{Conf}}_{A}=\frac{i}{i+j+k}=\text{Diff}\frac{i}{i-j}=\text{Diff}\frac{i}{\Delta }.$$ 10

Thus, the best-fitting model in Li and Ma (2020), which was used to argue against the Bayesian confidence hypothesis, is proportional to the predictions of the ABS using the max-minus-next stopping rule.

### Extending to Complex Representations

The behaviors discussed here are low-dimensional, with responses being situated within one- or two-dimensional spaces. As a result, the transformation from hypothesis samples to behavior is relatively simple, sometimes just with a linear mapping. But the scope and diversity of human behavior is much broader: many complex human behaviors are both high-dimensional and embedded in hierarchically organized spaces. Drawing, for example, or even copying line-drawings, requires sophisticated mental processes that represent a description of a drawing’s parts (e.g., lines and circles) and higher-order relations (e.g., repetition and hierarchy; Tian et al., 2020; Van Sommers, 1984). The motor system that implements routines and trajectories for turning these rich, structured representations into motor commands to produce drawings is also doing a more complex task than in low-dimensional behavior (e.g., complex trajectories may to be segmented into discrete, and hierarchically organized actions). Thus, the hypothesis space from which outputs are selected can be open-ended, and hierarchically organized at a range of levels of abstraction. While it is difficult to see how sensory accumulation models such as the SPRT or the DDM might extend to such cases, it is at least possible in principle to see how a Bayesian, sampling-based approach might operate. For example, many existing models of cognition, perception, and motor control involve Bayesian inference over compositional symbolic representations (e.g., Lake et al., 2017)—and the relevant computations can only be approximated, often using sampling (frequently, using standard MCMC). An interesting direction for future research is how far these Bayesian models can be mapped into fine-grained data relating detailed measures of high-dimensional output (including, for example, accuracy and variability) to fine-grained performance features, such as timing, and autocorrelations across trials. In general, where a Bayesian cognitive model can be defined, a sampling approximation to that model can be created, and compared with detailed process data from experiments. Thus, the ABS provides a possible bridge from simple, but intensively studied, decisions concerning binary choice or one-dimensional estimation, to models of cognition operating at full scale.

### Assessing the Rationality of the ABS

Bayesian models of cognition, pitched at Marr’s computational level, combines all available trial information (that is, prior knowledge of hypotheses, *p*(*h*), and the likelihood of the data presented in the trial given a hypothesis, p(h|s)) using the rules of probability theory. In doing so, Bayesian models fully extract trial information with 100% efficiency (Zellner, 2002).

We see the ABS as Bayesian in two ways. First, the ABS provides a sample-based approximation to an underlying Bayesian representation of a task. Combining the ABS with a Bayesian representation produces a rational process model (Griffiths et al., 2012). In other words, it is an algorithmic approximation of a computational-level model, which transforms it into a process model. A rational process model does not align perfectly with the underlying computational-level model because sampling approximations inevitably lead to loss of information. As a result, sampling models predict mistakes, systematic biases, and variability in behavior that are due to using stochastic samples.

Although we developed the ABS with a Bayesian perspective in mind, the underlying model does not necessarily have to be Bayesian. The probabilistic representation could be simply the relative frequencies of past events, without an associated probabilistic model (e.g., Costello & Watts, 2014). Alternatively, the probabilistic representation might not even be described as optimal or rational (Tauber et al., 2017). All that is required is that the representation can be written as a probability distribution, which covers a wide range of representations—after all, any finite set of nonnegative numbers can be normalized to become a probability distribution.

The second way in which the ABS is Bayesian is that the Bayesian Monte Carlo approach is used to interpret the samples that are generated by the model itself. This allows the ABS to take advantage of context-free expectations about the underlying probabilities, which improves probability (and confidence) judgments when a small number of samples give only imprecise information about those probabilities. We assume a conjugate prior on responses, making this process computationally very simple. Our analysis suggests that people do incorporate this prior on responses in forming behaviors—as we have seen, this assumption explains many classical empirical effects in probability judgments. Simple adaptivity in constructing the prior on responses (e.g., adapting to immediate feedback from the preceding trial) also helps explain human data such as fast errors.

This raises the question of whether the ABS makes optimal use of the sampling mechanism it has available. This is a different kind of normative concern than just fully extracting trial information. Here the question is whether the ABS is *resource rational* (Bhui et al., 2021; Lieder & Griffiths, 2020). Aside from the Bayesian Monte Carlo prior on responses, which could be argued to be resource rational, there is the smaller scale temporal tradeoff between either generating another sample which takes time or stopping and making do with the samples collected so far. The optimal stopping rule relies on solving a difficult dynamic programming problem (detailed in Appendix C). Indeed, given the fact that the optimal stopping rule is generally computationally intractable, we assumed another approximation to the optimal stopping rule with the max-minus-next rule, which has been independently suggested to well-approximate the performance of optimal stopping rule in the information theory literature (Dragalin et al., 1999). This optional stopping rule helps explain empirical patterns such as repulsion, slow errors, and resolution of confidence.

In short, we argue that exploiting algorithmic approximations to the optimal solution is the key feature of the ABS that justifies it as a rational process model. Our proposal, nonetheless, does not address the metalevel theoretical question concerning how the mind allocates cognitive resources across these approximation algorithms (e.g., the local sampler, the Bayesian Monte Carlo process, and the approximate dynamic-programming solution for stopping). A fully resource rational analysis would further specify the balance between the times spent on each approximation algorithm and the incentives from the task. Whether an optimal allocation of the limited cognitive resources is at play merits future investigations.

### The Prospects for Quantitatively Fitting the ABS

Quantitatively fitting a psychological model and determining meaningful parameter values are crucial for evaluating and comparing models. However, it can be challenging to apply standard fitting methods to the ABS when the assumptions of the model do not align with those of the fitting methods. We will elaborate the discrepancies in assumptions and provide suggestions for fitting methods that can be used to overcome these issues.

**Figure 12 fig12:**
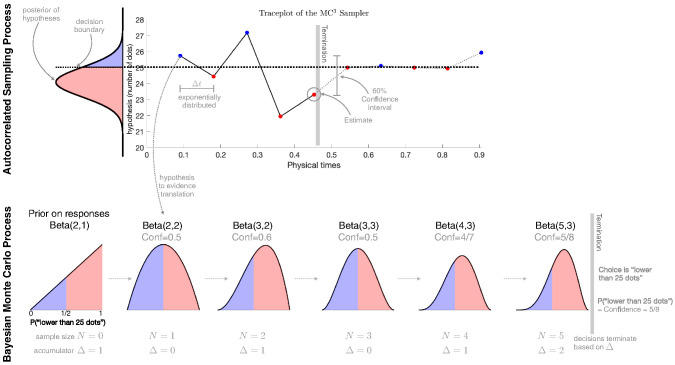
Further Illustrations of the Autocorrelated Sampling Process (Top) and the Bayesian Monte Carlo Process (Bottom), Expanding the Illustrative Example of Numerosity in Figure 2B *Note*. Here, the sampler was automatically terminated when five samples were generated, while the dashed lines denote potential future samples if continued. Samples were compared to a decision boundary of 25 (red dots: evidence for lower than 25 dots, blue dots: evidence for greater than or equal to 25). The five samples were then integrated with a prior on responses (here used an asymmetric prior, Beta(2, 1)), reaching a posterior of Beta(5, 3). The mean of this posterior on responses was then used to generate probability judgments or confidence judgments in decision-making. See the online article for the color version of this figure.

First, standard likelihood-based fitting methods, such as the maximum likelihood method, assume independence of behavioral data across trials, while the ABS assumes positive correlations both across and within trials, making it difficult to obtain a robust fit. Second, the ABS’s autocorrelated sampling process (as illustrated in Figure 12 top) is inherently stochastic, which means that even with the same inputs, predicted behaviors will not be identical. Moreover, there is no closed-form solution or accurate approximation of the distribution of the predicted behaviors under the ABS, preventing a closed-form likelihood function of behavioral data given the ABS.[Fig fig12]

One way to improve the robustness of the fit is to use a group of trials instead of individual trials. Grouping can be helpful because it makes the data more independent at the group level. Various methods such as quantiles can be used to group behavioral data. For example, Heathcote et al. (2002) have demonstrated that grouping RT data by sample quantiles produces a more efficient and less biased estimator. To take this method further, one could use likelihood-free techniques such as approximate Bayesian computation (ABC) as a more principled way to evaluate models with group-level data or any other summary statistics (Turner & Van Zandt, 2012). With the ABC method, we can simulate a series of behaviors predicted by the ABS using a set of parameter values, and compare the summary of the simulated behaviors with the summary of human behavioral data. Then we adjust the parameter values based on the similarity of the two summaries, and repeat the process until a satisfactory threshold is reached. The ABC method appear to be the most suitable fitting approach for our model due to its ability to handle issues such as autocorrelation and the absence of likelihood functions, as well as the ability to control for differing model flexibility. Some initial work has been done in fitting estimates using ABC (Spicer et al., 2022b; Zhu, León-Villagrá, et al., 2022) and relatedly fitting summary statistics of probability judgments using linear regression (Sundh et al., 2021). While in probability judgments the parameters of the prior are uniquely identifiable (Sundh et al., 2021), it would need to be established that the full range of model parameters are uniquely identifiable for the ABS to be used as a measurement model to interpret observed behavior in the way that DDMs are.

## Conclusions

We have outlined a rational process model of human behavior: the ABS. The ABS is rooted in a Bayesian framework, where the cognitive system is presumed to have an internal probabilistic model, which describes how the sensory data is generated in the real world. But rather than representing and computing with probabilities, we assume that the cognitive system uses a tractable approximation of the posterior which is realized via a local sampling algorithm. Distinct aspects of these posterior samples are relevant for different types of query and using simple and natural transformations, they provide a unified explanation of probability judgments, estimates, confidence intervals, choices, confidence judgments, and the time course of the posterior sampling accounts for RT.

Our framework shifts the locus of explanations for the accumulation of sensory input to computation (through sampling) over internal hypotheses. Thus, in our framework, the time course, and variability, of behavior is primarily explained in terms of an internal, noisy, computational process (involved in sampling from the hypothesis space), rather than through perfect Bayesian computation using noisy sensory data. We demonstrate the usefulness of our theory by reproducing key pair-wise relationships and stylized facts for the six behavioral measures and point the way toward extending the approach to the complex probabilistic models required to describe the richness of human behavior.

## Figures and Tables

**Table 1 tbl1:** Key Empirical Effects of the Six Behavioral Measures

Behavioral measures	Empirical effects	Description	Example references	ABS explanations
Probability judgments	Conservatism and probabilistic identities	Probability judgments show a linear bias toward 0.5 for binary events	Erev et al. (1994); Costello and Watts (2014)	Incorporating a symmetric prior on responses pulls probability judgments toward 0.5 for binary events
	Mean–variance relationship	The variance of probability judgments is highest for moderate probabilities and goes to zero before reaching the limits of the scale	Sundh et al. (2021)	Same as above
	Explicit subadditivity	When assessed separately, the sum of the probability judgments of the unpacked descriptors exceeds that of the packed descriptor	Tversky and Koehler (1994); Fischhoff et al. (1978); Olson (1976); Mehle et al. (1981); Russo and Kolzow (1994)	Same as above
	Conjunction fallacy	The judged probability of the conjunctive event is higher than that of its constituents	Tversky and Kahneman, (1983); Costello and Watts (2014)	Because of their added complexity, fewer conjunctive event samples are generated, resulting in a greater pull toward 0.5 for binary events
	Implicit subadditivity in typical unpacking	When assessed jointly, the probability judgment of the unpacked descriptor exceeds that of the packed descriptor when the unpacked descriptor includes high probability events	Sloman et al. (2004); Dasgupta et al. (2017)	The hypothesis that the autocorrelated sampler initializes on tends to be probable (i.e., typical examples)
	Implicit superadditivity in atypical unpacking	When assessed jointly, the probability judgment of the unpacked descriptor is smaller than that of the packed descriptor when the unpacked descriptor includes low probability events	Sloman et al. (2004); Dasgupta et al. (2017)	The hypothesis that the autocorrelated sampler initializes on tends to be improbable (i.e., atypical examples)
	Partition dependence	Probability judgments are biased toward an even allocation across all possible events	Fox and Rottenstreich (2003); Attneave (1953); Bardolet et al. (2011); Varey et al. (1990)	Incorporating a symmetric Dirichlet prior on responses (i.e., a multivariate generalization of the Beta prior) pulls probability judgments toward 1/*M* for *M*-alternative events
Decisions affecting later responses	Anchoring	Estimates are pulled toward the boundary used in the preceding decision when the boundary is far from the correct answer	Tversky and Kahneman (1974); Epley and Gilovich (2006); Spicer et al. (2022a)	The initial hypothesis used by the autocorrelated sampler will often be the comparison value used in the decision task (i.e., the anchored hypothesis). Moreover, the samples used to reach the decision are assumed to be reused in the estimation task
	Repulsion	Estimates are pushed away from the boundary used in the preceding decision when the boundary is close to the correct answer	Jazayeri and Movshon (2007); Luu and Stocker (2018); Spicer et al. (2022a)	The optional stopping rule only terminates the sampler whenever sufficiently more evidence supporting one alternative is obtained. As above, samples used to reach the decision are reused in the estimation task
Accuracy and response times	Speed–accuracy trade-off	More accurate decisions are associated with longer decision times such that people can trade accuracy for speed	Garrett (1922); Johnson (1939)	The sequential arrival of samples predicts that, on average, a greater sample size requires more time to generate but results in more accurate choices. This tradeoff is controlled by the decision threshold, in which people can balance the cost of waiting and the benefits of a refined approximation by adjusting the threshold
	Slow errors	For difficult conditions and when accuracy is emphasized, mean decision times for incorrect choices are slower than those for correct choices	Ratcliff and Rouder (1998); Vickers (1979); Townsend and Ashby (1983)	Optionally terminating an autocorrelated sampling process will increase the proportions of slower responses for error responses (vice versa for correct responses)
	Fast errors	For easy conditions and when speed is emphasized, mean decision times for incorrect choices are faster than those for correct choices	Ratcliff and Rouder (1998); Townsend and Ashby (1983)	Adaptively updating the prior on responses to reflect previous choice outcomes will increase the proportions for fast responses that are errors
	Near-linear relationship of RT quantiles with a fan shape	The quantiles of the RTs from two difficulty levels are close to linear	Ratcliff et al. (2003); Ratcliff et al. (2015)	Optionally terminating an autocorrelated sampling process and the waiting time between two consecutive samples being exponentially distributed make the tails of RT distributions more spread out for harder tasks
Confidence in decisions	Positive relationship between confidence and stimuli discriminability	Confidence increases as stimuli discriminability increases	Baranski and Petrusic (1998); Garrett (1922); Vickers (1979); Johnson (1939); Vickers and Packer (1982)	Higher stimuli discriminability partitions the hypothesis space to more unevenly favor the correct response. Thus, the hypothesis samples that were converted into evidence become more homogenous with increasing discriminability
	Resolution of confidence	There is a positive relationship between choice accuracy and confidence judgments	Baranski and Petrusic (1998); Vickers (1979); Garrett (1922); Ariely et al. (2000); Johnson (1939); Vickers and Packer (1982)	Optionally terminating an autocorrelated sampling process will make the correct responses faster and thereby greater confidences
	Metacognitive inefficiency	Metacognitive efficiency (as measured by *meta_d*′/*d*′) decreases for higher confidence ratings	Shekhar and Rahnev (2021a, 2021b)	Confidence is based on the proportion of response-consistent binomial samples (see Appendix D, for detail). The sample autocorrelation amplifies the metacognitive inefficiency
	Negative (cross-trial) relationship between confidence and RT	Within the same condition, confidence decreases as RT increases	Henmon (1911); Baranski and Petrusic (1998); Vickers and Packer (1982); Johnson (1939)	Confidence is based on the proportion of response-consistent samples with an optional stopping rule
	Positive (cross-condition) relationship between confidence and RT	People are more confident in conditions in which they take more time to make a choice	Vickers and Packer (1982)	The decision threshold is greater in the accuracy condition than in the speed condition, so choices will take longer while the greater difference in sample counts at threshold leads to higher confidence
Confidence intervals	Strong overconfidence in self-produced confidence intervals	Self-generated confidence intervals are far too narrow	Juslin et al. (2007); Juslin et al. (2003); Juslin and Persson (2002)	Sample quantiles are too narrow to represent distributional intervals
	Little or no overconfidence in evaluation of confidence intervals	Evaluations of confidence intervals are well calibrated	Juslin et al. (2007); Juslin et al. (2003); Juslin and Persson (2002)	Samples are relatively representative of the target distribution on aggregate
Cross-trial autocorrelation in RT and estimates	Long-range autocorrelations in estimation time series	Sequences of estimates show autocorrelations that are proportional to frequency (1/*f* noise)	Gilden et al. (1995); Gilden (2001)	The autocorrelations are generated by the local nature of sampling
	Lesser long-range autocorrelations in RT time series	Sequences of RTs also show a certain degree of autocorrelation but are closer to independent than are sequences of estimates	Wagenmakers et al. (2004)	Basing RT on the number of autocorrelated samples until threshold (i.e., counting autocorrelated samples) reduces the degree of autocorrelation in samples
*Note*. ABS = Autocorrelated Bayesian Sampler; RT = response times.				

**Table 2 tbl2:** Which Models Reproduce the Key Empirical Targets

Behavioral measures	Empirical effects	SPRT	Bayesian sampler (Zhu et al., 2020)	Autocorrelated Bayesian Sampler			
				No prior variant	Direct sampling variant	Fixed sample size variant	Full model
Probability judgments	Conservatism and probabilistic identities	N/A	✓	✗	✓	✓	✓
	Mean-variance relationship	N/A	✓	✗	✓	✓	✓
	Explicit subadditivity	N/A	✓	✗	✓	✓	✓
	Conjunction fallacy	N/A	✓	✗	✓	✓	✓
	Implicit subadditivity in typical unpacking	N/A	✗	✓	✗	✓	✓
	Implicit superadditivity in atypical unpacking	N/A	✗	✓	✗	✓	✓
	Partition dependence	N/A	✓	✗	✓	✓	✓
Decisions affecting later responses	Anchoring	N/A	N/A	✓	✗	✓	✓
	Repulsion	N/A	N/A	✓	✓	✗	✓
Accuracy and response times	Speed–accuracy trade-off	✓	N/A	✓	✓	✓	✓
	Slow errors	✗	N/A	✓	✗	✗	✓
	Fast errors	✗	N/A	✗	✓	✗	✓
	Near-linear relationship of RT quantiles with a fan shape	✓	N/A	✓	✓	✗	✓
Confidence in decisions	Positive relationship between confidence and stimulus discriminability	✓	N/A	✓	✓	✓	✓
	Resolution of confidence	✗	N/A	✓	✗	✗	✓
	Metacognitive inefficiency	✗	N/A	✓	✓	✓	✓
	Negative (cross-trials) relationship between confidence and RT	✗	N/A	✓	✓	✗	✓
	Positive (cross-conditions) relationship between confidence and RT	✓	N/A	✓	✓	✓	✓
Confidence intervals	Strong overconfidence in self-produced confidence intervals	N/A	N/A	✓	✓	✓	✓
	Little or no overconfidence in evaluation of confidence intervals	N/A	N/A	✓	✓	✓	✓
Cross-trial autocorrelation in RT and estimates	Large long-range autocorrelation in estimation time series	N/A	N/A	✓	✗	✓	✓
	Lesser long-range autocorrelation in RT time series	✗	N/A	✓	✗	✗	✓
*Note*. N/A denotes that the model is nonapplicable to the empirical effect because it does not produce the relevant behavioral measure. SPRT = sequential probability ratio test; RT = response times.							

**Figure C1 fig13:**
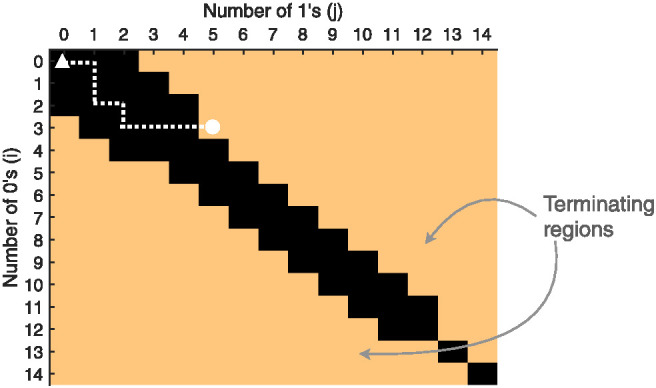
Decision Thresholds Obtained From Solving the Dynamic Programming Problem *Note*. The sampling algorithm should be terminated once the accumulator reaches the yellow terminating regions. In this illustration, the accumulator started at the top-left corner {*i* = 0, *j* = 0} (white triangle) and terminated at the state {*i* = 3, *j* = 5} (white circle). See the online article for the color version of this figure.

**Figure D1 fig14:**
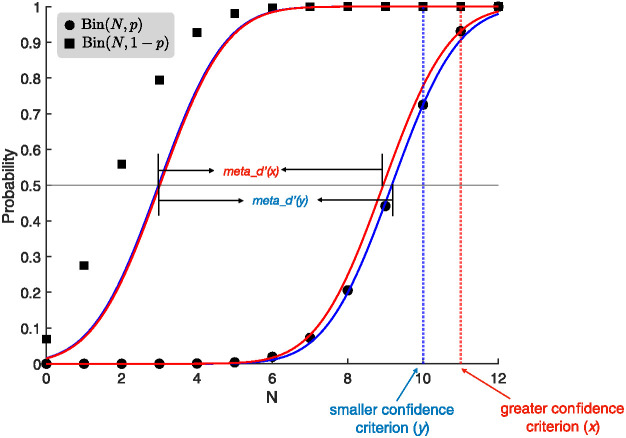
An Illustration of Metacognitive Inefficiency Arising Not From Loss of Information in Confidence Judgment, but From Incorrectly Assuming Gaussian Generating Distributions *Note*. Two confidence criteria were shown (*x*, *y*, where *x* > *y*). Hit rates were calculated based on the binomial distribution Bin(*N,p*) (black circles) given its intersection with a confidence criterion, whereas similarly false alarm rates were calculated using the symmetric binomial distribution Bin(*N*, 1 − *p*) (black squares). In this illustrative example, *N* = 12 and *p* = .8. Using a Gaussian to compute *mete_d*′ (difference in the horizontal positions of the solid curves) will lead to a decrease in value when the confidence criterion increases: *mete_d*′(*x*) < *mete_d*′(*y*). See the online article for the color version of this figure.

**Figure E1 fig15:**
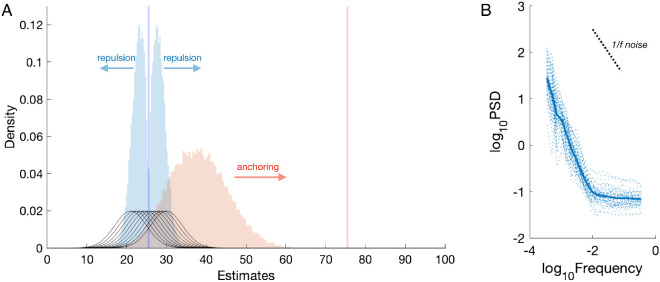
Using the Sample Average, Rather Than the Last Sample, as the Estimate Does Not Qualitatively Change Model Behaviors *Note*. (A) The model predicts co-occurrence of repulsion and anchoring as shown in Figure 10. (B) The model produces 1/*f* noise as shown in Figure 11. The sample size used in the simulations was fixed at 5. See the online article for the color version of this figure.

## Footnotes

1 We will only occasionally touch on the large and distinct literature on risky decision-making based on verbally or numerically problems in the fields of judgment and decision-making and behavioral economics (e.g., Tversky & Kahneman, 1974), which has largely developed as a separate tradition.

2 We do not further consider other general-purpose approximation algorithms, such as variational inference, in which a simpler distribution is used to approximate a more complex one, and the statistical distances between the two distributions are minimized by optimization algorithms. Such algorithms provide an alternative source of explanations for human behavior (Gershman & Beck, 2017; Sanborn, 2017).

3 Note that this prior reflects the prior knowledge of the hypothesis space and therefore should be amenable to feedback and experimental instructions about the hypothesis space. For the running numerosity example, the experimenter could explicitly inform participants, or they could learn through experiences, that the numbers of dots appearing on-screen across trials are uniformly distributed in the range of [21, 30]. Indeed, previous work has shown that participants can quickly acquire an accurate prior from feedback in a numerosity task (Sanborn & Beierholm, 2016). In this case, for simplicity, we should use a uniform distribution as the prior for hypotheses.

4 People often use round numbers when making estimates, and indeed typically focus on so-called prominent numbers (Converse & Dennis, 2018). There is evidence that round numbers are used to communicate confidence in the estimate (Jerez-Fernandez et al., 2014; Welsh et al., 2011) and round number use is reduced when responses are made on a continuous scale (Honda et al., 2022). Future work could explore adding a rounding mechanism for this purpose.

5 Nondecision time, as is commonly used in models of response time, would be necessary to include to quantitatively reproduce empirical RT distributions, but it is not needed to produce the qualitative effects we evaluate below.

6 The Bayesian Monte Carlo prior on responses is different from the prior on hypothesis in Equation 3. Specifically, the Bayesian Monte Carlo prior should capture full or partial information about the frequencies of the relevant behavioral outcomes in past trials. Consider again the binary choice in the numerosity example where people were asked to judge whether the number of dots is greater than 25. In this case, the prior on responses should reflect, to some extent, prior belief in different probabilities that each response is correct; and this prior knowledge could be acquired through feedback of correctly choosing greater-than-25 and of correctly choosing the alternative less-or-equal-than-25. An additional difference is that when knowledge of the probabilities is precise, even if there is uncertainty in the hypotheses, the effect of the Bayesian Monte Carlo prior reduces. Thus, in Equation 5, as the sample size, *N*, approaches infinity then $\hat{P}(A)$ tends to *P*(*A*).

7 Incorporating historical information into new situations is known as power prior in the statistics literature, which is also closely related to the ideas of metalearning (or learning-to-learn) and hierarchical Bayesian modeling.

8 Confidence judgments are often made on various ordinal rather than probability scales, though analyses of confidence judgments often just assume that they are monotonically related (Li & Ma, 2020; Shekhar & Rahnev, 2021a), as we do here. For comparability across different ordinal scales we present all of the model predictions on the probability scale rather than specifying those relationships.

9 A reanalysis of the four probability judgment experiments presented in Sundh et al. (2021) shows that participants produced indifferent probability judgments (i.e., exactly 50 on a 0–100 scale) on about 6% of trials.

10 The autocorrelation of the samples does not qualitatively alter the overall model predictions for probability judgments (with the exception of implicit subadditivity and implicit superadditivity, discussed below) because autocorrelated sample sizes can be corrected to produce the effective sample size of independent samples using the following equation: $N_{\text{eff}}=\frac{N}{1+2\sum_{t=1}^{\infty }\rho_{t}}$ where ρ_*t*_ is the degree of autocorrelation at lag *t*. For the parameters we used in the simulations, the effective sample size is on average 16.80% of the autocorrelated sample size (95% CI [16.40%, 17.21%]). In addition, in the studies we refer to, there is very rarely any feedback. Without feedback, we assume the ABS prior does not change from trial to trial, making it identical to the fixed prior of the Bayesian Sampler for binary events.

11 A reduction in trial difficulty can arise due to a less variable posterior of hypotheses (e.g., people are more certain about the number of dots), or because the decision boundary that partitions the hypothesis space is at one extreme (e.g., people are asked to judge whether the number of dots are less than 1,000 while only 24 dots appeared on-screen), or both. Both factors contribute to an increase in the proportion of samples that support the correct response.

12 While this characterizes an SPRT that stops at the threshold (e.g., as discussed in Pleskac & Busemeyer, 2010; Vickers, 1979), there are variants that would make different predictions. For example, the boundaries do not necessarily have to be symmetric, or they could collapse. Also, it is possible that the SPRT’s confidence would reflect not just the threshold but the posterior probability of all the sensory samples that were observed before stopping, with confidence then being greater than or equal to the threshold confidence. All these variants would produce variable posterior probabilities and hence variable confidence judgments. However, none of these variants would produce estimates, confidence intervals, or other judgments that are generated by the ABS, but which are beyond the scope of the SPRT.

13 Informativeness of choices and confidence ratings are measured as stimulus sensitivity *d*′ and *meta_d*′ respectively (Fleming & Lau, 2014; Maniscalco & Lau, 2012). More specifically, *d*′ = ϕ^−1^(hit rate) − ϕ^−1^(false alarm rate) where ϕ^−1^ is the inverse of the cumulative Gaussian distribution. *meta_d*′ is calculated in the same manner but with the hit rate and the false alarm rate tallied according to a criterion value that partitions confidence ratings. More specifically, the hit rate is the proportion of trials in which participants reported high confidence given a correct response, whereas the false alarm rate is the proportion of trials in which participants reported high confidence given an incorrect response; and the confidence criterion value determines whether a confidence judgment is considered high or low.

14 There is only slight overconfidence predicted by the naïve intuitive statistican model when the internal generative model does not perfectly describe the data generating process (Juslin et al., 2007), and the ABS would show the same effect with an imperfect model of the data generating process.

15 1/*f* noise goes under various names in different literatures (e.g., pink noise and flicker noise). Besides cognition, diverse complex processes have also been found to exhibit this type of long-range dependence. For example, magnetoencephalography and electroencephalogram data from human brains, and indeed classical music, all exhibit 1/*f*-like fluctuations (Linkenkaer-Hansen et al., 2001; Novikov et al., 1997).

16 Regardless of trial-by-trial variability in drift-rates and/or starting points.

17 Alternatively, without changing the absorbing threshold, we can adjust the drift rate to mimic a sampling algorithm. The idea is that a diffusing particle that moves with a bias in the direction of higher probability can be linked to local sampling algorithms (Ma et al., 2019; Roberts & Stramer, 2002; Rossky et al., 1978). Indeed, local samplers move around the space stochastically, which can be described as a diffusion process. Therefore, by carefully designing the biasing method for diffusion, the long-term behavior will converge to as if drawing samples from a distribution. For instance, a class of Lengevin diffusions has been designed such that the stationary distribution of the diffusion is in proportion to the target distribution of MCMC sampling (Ma et al., 2019; Roberts & Stramer, 2002).

## A Algorithm and Simulation Details

Throughout the article, we used a consistent set of parameters in simulating the Autocorrelated Bayesian Sampler. All simulations were repeated 2^14^ times. The target posterior distributions (i.e., P(h|s) in Equation 3) for sampling were Gaussian distributions: *N*(μ = 0, σ = 1) for choice tasks and *N*(μ = μ_target_, σ = 4) for estimation tasks. The stimuli discriminability was manipulated at five different values by fully randomizing the decision boundaries between {−0.2, 0.2} (for the least discriminable stimuli), {−0.5, 0.5}, {−0.7, 0.7}, {−1, 1}, and {−2, 2} (for the most discriminable stimuli). When accuracy was emphasized, we set a larger value for decision threshold Δ = 6, whereas we set Δ = 2 when speed was emphasized. To qualitatively reflect that increasing visual contrasts reduces task difficulty, as shown in Figure 8B, we simulated Gaussian target distributions with means all equal to 0 but standard deviations of 1.5 (Contrast 1), 1.3 (Contrast 2), and 1 (Contrast 3).

We simulated a local sampling algorithm, MC^3^, which draws samples from the unnormalized posterior (Geyer, 1991). MC^3^ runs parallel Markov chains at different temperatures and allows individual chains to exchange information. We fixed the number of parallel chains at *C* = 6 and varied the temperature between the chains where the *c*-th chain has a temperature of $T_{c}=\frac{1}{1+4(c-1)}$. The higher temperature chains will draw samples from flattened target distributions, thus prefer making long distance moves and assist lower temperature chains to make nonlocal jumps through the swapping step (see Algorithm, for details). This means that the first chain is also the cold chain (temperature of 1) such that its target distribution is not heated up and thus undistorted. The widths of proposal distribution were always in smaller proportions to the width of target distribution, where the ratios were set at 1:100 for choice tasks and 1:1.5 for estimation tasks. The average speed of generating a sample λ = 0.1 s^−1^.

Algorithm Metropolis-coupled Markov chain Monte Carlo (MC3)
Choose a starting hypothesis h1, target unnormalized posterior ϕ(h) = P*(h|s)
for n=1 to N ⊳generate N samples
for c=1 to C ⊳update all C chains
Draw a candidate hypothesis h′∼𝒩(hcn−1, σ2) ⊳Gaussian proposal
Ac = min{1, [ϕ(h′)/ϕ(hcn−1)]1/Tc} ⊳ acceptance probability
Sample u∼U[0,1] ⊳ acceptance threshold
if u < Ac then hcn = h′ else hcn = hcn−1 end if ⊳ decide acceptance
end for
repeat floor(C/2) times ⊳ chain swapping
Randomly select two chains i, j without repetition
Aswap = min{1,ϕ(hjn)1/Tiϕ(hin)1/Tj/ϕ(hin)1/Tiϕ(hjn)1/Tj} ⊳ swapping probability
Sample u∼U[0,1] ⊳ swapping threshold
if u < Aswap then swap(hin, hjn) end if ⊳ decide swapping
end repeat
end for

## B Reinterpretations of Nosofsky and Palmeri’s (1997) Exemplar-Based Random Walk Model and Blurton et al. (2020) Poisson Random Walk Model

In this section, we provide detailed reinterpretations of Nosofsky and Palmeri’s (1997) EBRW model and Blurton et al. (2020) PRW model. This analysis helps shed light on the relationship between the ABS and these existing computational models. In short, the direct sampling variant of the ABS parallels the model mechanisms of both the EBRW and PRW models.

### Nosofsky and Palmeri’s (1997) Exemplar-Based Random Walk Model

The EBRW model, a generalization of Logan’s instance-based model (Logan, 1988), aims to explain the relationship between categorization and response times by integrating exemplar-based models of categorization and response–time models of evidence accumulation (Nosofsky & Palmeri, 1997). Exemplar models assume that people store many instances (“exemplars”) of events in memory, and evaluate new events by activating stored exemplars according to their similarity (Medin & Schaffer, 1978; Nosofsky, 1986). This idea has been formalized in the Generalized Context Model (Nosofsky, 1986): individual exemplars are assumed to be represented as points in a multidimensional psychological space where similarity between exemplars decreases with the distance between objects in the space (Shepard, 1987). For a new observation *x* and a set of *K* stored exemplars $X^{*}=\{x_{1}^{*},\;x_{2}^{*},\cdots ,\;x_{K}^{*}\}$, all exemplars are activated in proportion to their similarity with the new observation *s*(*x*, *x**). The predictive value for the new observation is:

$$\tilde{f}(x)=\frac{\sum_{i=1}^{K}f\left(x_{i}\right)s\left(x,\;x_{i}\right)}{\sum_{i=1}^{K}s\left(x,\;x_{i}\right)},$$ B1

where *f*(*x*_*i*_) is the information associated with the exemplar *x*_*i*_. This equation is a general expression for many cognitive applications of the exemplar model. For example, to identify *x* within the set of exemplars *X** (i.e., $\tilde{f}(x)=p(x^{*}|x)),$ we can set the function $f(x_{i})=\{\begin{array}{c} 1\;\text{if}\;x_{i}=x\\ 0\;\text{otherwise} \end{array}$; similarly, to judge the probability of *x* belonging to a category *c* (i.e., $\tilde{f}(x)=p(c|x)$), $f(x_{i})=\{\begin{array}{c} 1\;\text{if}\;c_{i}=c\\ 0\;\text{otherwise} \end{array}$.

When the function *f*(*x*_*i*_) is an indicator function as it was used in the categorization tasks, the resultant $\tilde{f}(x)$ is a discrete probability distribution within a continuous psychological space. The probability associated with the *i*-th exemplar equals $\frac{s\left(x,x_{i}\right)}{\sum_{i=1}^{K}s\left(x,x_{i}\right)}$ if its indicator function takes a value of 1. In this case, the exemplar-based model provides one way to construct the posterior probability of hypotheses by incorporating similarity-weighted information from stored exemplars. EBRW then supposes that people sequentially draw samples according to $\tilde{f}(x)$ until a threshold number of samples in favor of a response is reached. The waiting times between samples are exponentially distributed, as in the ABS, though each waiting time also includes an additive constant which is not currently present in our model. Thus, the EBRW is nearly equivalent to the direct sampling variant of the ABS in which there is no interdependence between samples of exemplars. What the full ABS can add to this model are autocorrelations between samples and an adaptive prior, which potentially could allow it to account for a wider range of behavior.

### Blurton et al.’s (2020) Poisson Random Walk Model

While closely related to the EBRW, Blurton et al.’s (2020) PRW model instead focuses on assigning confusable visual stimuli to perceptual categories (e.g., whether a Gabor patch is oriented to the left or right of vertical) and is based on the Theory of Visual Attention (Bundesen, 1990). The TVA’s contribution can be captured by Equation B1, with the additional assumption of PRW being that the interstep time between two successive tentative categories is exponentially distributed with a rate *C*. Thus, for *N* samples generated in this way, the response time should follow an Erlang distribution (the sum of *N* exponentially distributed intervals):

f(t|N, C)=Erlang(N, C), B2

where, in the binary case, the average speed of generating a tentative category *C* = *v*_*A*_ + *v*_*B*_ where *v*_*A*_, *v*_*B*_ represent the strengths of category *A*, *B* respectively. In other words, the probability of a tentative category being *A* is $p_{A}=\frac{v_{A}}{v_{A}+v_{B}}$ and that of being *B* is $p_{B}=1-p_{A}=\frac{v_{B}}{v_{A}+v_{B}}$. If we consider the category *A* as the upper bound and the category *B* as the lower bound, the random walk of the accumulator has an increment of +1 with probability *p*_*A*_ and −1 with probability *p*_*B*_. Coupling the Poisson process of tentative category generation with an optional stopping rule whose accumulator starts at *z* (0 < *z* < *a*) and evidence thresholds *k*_*A*_ = *a*, *k*_*B*_ = 0, we can derive the cumulative distribution function of first passage time to state *k*_*B*_ (Equation A5 of Blurton et al., 2020):

$$F_{B}(t|v_{A},\;v_{B},\;a,\;z)=\sum_{n=z}^{\infty }F(t|n,\;v_{A}+v_{B})f_{B}(n|\frac{v_{A}}{v_{A}+v_{B}},\;a,\;z),$$ B3

where $F(t|n,v_{A}+v_{B})$ is the cumulative distribution function of the Erlang distribution above. Because of the fixed thresholds (0, *a*) and starting point (*z*), the number of samples (or number of increments) in the random walk to reach one of the thresholds is a random variable. *n* can be as small as *z* (for reaching threshold *B*) or *a* − *z* (for reaching threshold *A*) if all the tentative categories generated in the process are for the same category. But due to the randomness in generating tentative categories, *n* can also be far greater than those numbers and the probability of *n* has a closed form solution (Blurton et al., 2020; Feller, 1968 Equation A1). Marginalizing over all possible *n*s produces the cumulative distribution function (CDF) of RT.

This basic version of the PRW is mathematically very close to both the decision process of the EBRW (as noted in Blurton et al., 2020) and a restricted version of the ABS that uses direct sampling and no adaptive prior. First, we can interpret *p*_*A*_, *p*_*B*_ as posterior probabilities of category *A* and *B* respectively and they also normalize to 1. Second, the Erlang distribution of RT given *N* samples is also assumed in the ABS (compare Equations 6 and B2). Third, the random walk process with an increment of +/−1 is equivalent to the heuristic stopping rule used in the ABS where the differences in number of samples is tracked. While *p*_*A*_, *p*_*B*_, *z* are free parameters for the PRW model, our reinterpretation will constrain their values. *p*_*A*_, *p*_*B*_ as the posterior probability should be further constrained by the mental representation of stimuli.

Beyond this basic formulation, the PRW also includes extensions to improve its fit to the data and its scope. To produce fast or slow average errors (in comparison to average correct responses), the PRW incorporates, following the approach used in DDMs, trial-by-trial variability in category strengths and starting points. To explain the variability in the leading edge of response time distributions, the PRW allows for processing rates to vary within a trial. The full version of the ABS produces fast or slow errors because of an adaptive prior and autocorrelated sampling respectively. While we did not explore the ABS’s predictions for variability in the leading edge of response time distributions, it would be interesting to see if autocorrelated sampling—effectively varying processing rates within a trial—produces this effect as well, or whether other mechanisms, such as a variable nondecision time, are needed.

The PRW also has been extended beyond two-alternative choice tasks to any number of alternatives by assuming that evidence for one response suppresses evidence for all other responses. The suppression is strong enough that only one counter has nonzero counts at any one time. This extension differs from our proposal in the Discussion of using a max-minus-next stopping rule for multialternative choice as there is no inhibition, but future work is needed to explore what contrasting predictions these mechanisms make.

## C Optimal Stopping Rule Is Approximated by the Heuristic Stopping Rule

The optimal solution to terminate the sampling algorithm is to solve the following dynamic programming problem. To illustrate, we demonstrate the optimal stopping rule in binary choice. Let *p*_00_ denote the prior probability of one alternative being true (i.e., the prior on responses) and *p*_*ij*_ the posterior probability after *i* 0 s and *j* 1 s have been accumulated. The posterior probability that the other alternative is true is simply 1 − *p*_*ij*_. The binary decision task is equivalent to a sequential statistical test on whether *p*_*ij*_ < 1/2, and the optimal stopping rule for this test can be formally described using dynamic programming as follows (Wald, 1949; Zhu et al., 2019):

$$F(i,\;\;j)=\min\{F_{0}(i,\;\;j),\;c+p_{ij}F(i,\;\;j+1)+(1-p_{ij})F(i+1,\;\;j)\},$$ C1

where *F*(*i*, *j*) and *F*0(*i*, *j*) are, respectively, expected cost of sampling and expected cost of termination after *i* samples against and *j* samples in favor have been observed, and *c* is the opportunity cost of generating a sample. Suppose that *p*_00_ = Beta(0, 0), then the posterior of evidence is also Beta distributed *p*_*ij*_ = Beta(*i*, *j*). If the punishment for an incorrect decision is one unit of utility, the expected cost of termination should be the expected cost of incorrectly choosing an alternative when posterior of that alternative is *p*_*ij*_:

$$F_{0}(i,\;\;j)=\min\{\frac{i}{i+j},\frac{j}{i+j}\}.$$ C2

The expected cost of drawing another sample is the sum of (a) the cost of generating one sample, *c*, (b) the expected cost if the new sample turns out to be in favor, *p*_*ij*_*F*(*i*, *j* + 1), and (c) the expected cost if the new sample turns out to be against, (1 − *p*_*ij*_)*F*(*i* + 1, *j*). The sampling process should stop whenever the expected cost of sampling exceeds that of termination: *F*(*i*, *j*) ≥ *F*_0_(*i*, *j*). We solved the dynamic programming and visualized its stopping rules in Figure C1 by setting the cost of collecting one sample, *c* = 0.006, and the prior probability of evidence to Beta(1, 1). While the optimal decision threshold is collapsing over time, it can be approximated by a heuristic max-minus-next stopping rule. Indeed, in multialternative settings, it has been shown that implementing the max-minus-next rule closely matches the performance of an optimal stopping rule (Dragalin et al., 1999; 2000).[Fig fig13]

## D Confidence From Binomial Sample Counts Can Produce Metacognitive Inefficiency

In this section, we demonstrate the problem that when confidence judgments are assumed to be tally of discrete sample counts (especially when the sample size is small), the so-called “metacognitive inefficiency” effect demonstrated by Shekhar and Rahnev (2021a) can also be present even without any loss of information (i.e., noise) in forming confidence judgments. To recap, metacognitive inefficiency describes a relationship between an informativeness measure (*mete_d*′/*d*′) and the confidence criterion which classifies confidence judgments as either high or low (Shekhar & Rahnev, 2021a). Both *mete_d*′ and *d*′ are model-based measures of informativeness that crucially assume Gaussian distributions (Fleming & Lau, 2014; Maniscalco & Lau, 2012). More specifically, the inverse Gaussian CDF is used to calculate *mete_d*′ and *d*′:

$$\text{met}a\_d^{\prime }(c)=\phi^{-1}(\text{hit rate}|c)-\phi^{-1}(\text{false alarm rate}|c),$$

where ϕ^−1^ is the inverse Gaussian CDF and *c* is the confidence criterion for which individual confidence judgments that are greater (smaller) than *c* are considered high (low). Note that when *c* = 50% (i.e., when choosing either option always receives the same level of confidence), *d*′ = *mete_d*′.

The empirical results uncovered using this approach indicate that as the confidence criterion (*c*) increases, *mete_d*′/*d*′ decreases (Shekhar & Rahnev, 2021a). As *d*′ is fixed in this calculation, that means that as *c* moves further into the upper tails, *mete_d*′(*c*) becomes smaller. This result has been used to argue that a certain amount of information about a decision was lost in the process of forming metacognitive judgments and that more extreme confidence judgments are noisier (Shekhar & Rahnev, 2021a, 2021b).

Here, we do not challenge the empirical validity of the effect. Instead, we show that assuming a Gaussian distribution when calculating the informativeness of metacognitive judgment can lead to a decrease in the measured metacognitive efficiency when there is no loss of information but the data-generating distribution is not Gaussian. To illustrate, we present an example when the data is generated by binomial distributions (see Figure D1). In this example, the cumulative Gaussian distributions that respectively intersect with the hit and false alarm distributions (which are cumulative binomial) become closer together as the confidence criterion becomes more extreme: *mete_d*′(*x*) < *mete_d*′(*y*) and *x* > *y* ≥ *Np*. The quantitative difference between the two *mete_d*′ of 0.16 is small but noticeable in this example (see Figure D1). However, this bias becomes smaller when *N* is large because the data-generating binomial distribution is better approximated by a Gaussian distribution.[Fig fig14]

## E ABS Predictions When Estimates are the Sample Average Instead of the Last Sample

Here we consider an alternative process of making estimates using sample averages instead of taking the last sample as the estimate. As shown in Figure E1, with this new process of producing estimates, the qualitative model behavior of the ABS was reproduced. Using the sample average as the estimate, however, does make the anchoring effect stronger, while repulsion and the degree of autocorrelation become weaker. This is because, compared to taking the last sample, averaging of all samples increases the impact of the earlier samples generated by the autocorrelated sampler.[Fig fig15]

Using the last sample as the estimate also implies that estimates will often fall outside the range of participant-produced confidence intervals, much more often than when using the mean or the median as the estimate. These differing predictions can be tested in future empirical work to distinguish between them.

## F The Process and Time for Producing Probability Judgment

In this section, we provide further justification for the ABS process of probability judgment in which (a) a fixed number of samples is collected and (b) the waiting time between two consecutive samples are exponentially distributed. The fixed-sample-size rule was assumed both for simplicity and because of the lack of an obvious target for deriving an optimal stopping rule for probability judgments. As shown in the main text, this process should produce Gamma-distributed RTs (i.e., continuous generalization of the Erlang distribution), which are unimodal and positively skewed. In Figure F1, this prediction is confirmed by the RT data collected from three experiments (Sundh et al., 2021; Zhu et al., 2020). The best-fitting shape and scale parameters for Experiments 1, 2, and 3 are Gamma(2.36, 3.43), Gamma(2.37, 3.97), and Gamma(2.22, 3.68) respectively. The RT distributions resemble each other despite being based on different groups of participants and different probability judgments, suggesting that people may indeed use a fixed-sample-size rule to produce probability judgments.[Fig fig16]

Alternatively, one could replace the fixed-sample-size rule with an optional stopping rule (e.g., the max-minus-next stopping rule) or a fixed-time stopping rule (e.g., stop after thinking about the task for 10 sec). A max-minus-next stopping rule would predict a mixture of Erlang distributions but would also predict no indifferent probability judgments because sampling continues under this rule until probability judgments are no longer indifferent. Furthermore, the max-minus-next stopping rule should predict that the probability judgments become less extreme, on average, for longer RTs. These predictions mismatch empirical data as discussed earlier and the empirical relationship between probability judgments and RTs shown in Figure F1A. On the other hand, a fixed-time rule predicts a Gaussian distribution of RTs if we further assume Gaussian noise in the estimation of elapsed time. This prediction should be rejected given the skewness in the RT distributions (see Figure F1B).

## References

[ref1] Abbott J. T., & Griffiths T. L. (2011). Exploring the influence of particle filter parameters on order effects in causal learning. In Carlson L., Hoelscher C., & Shipley T. F. (Eds.), Proceedings of the annual meeting of the cognitive science society (pp. 2950–2955). Cognitive Science Society.

[ref2] Anderson J. R. (1991). The adaptive nature of human categorization. Psychological Review, 98(3), 409–429. 10.1037/0033-295X.98.3.409

[ref3] Andrieu C., De Freitas N., Doucet A., & Jordan M. I. (2003). An introduction to MCMC for machine learning. Machine Learning, 50(1), 5–43. 10.1023/A:1020281327116

[ref4] Aragones E., Gilboa I., Postlewaite A., & Schmeidler D. (2005). Fact-free learning. The American Economic Review, 95(5), 1355–1368. 10.1257/000282805775014308

[ref5] Ariely D., Au W. T., Bender R. H., Budescu D. V., Dietz C. B., Gu H., Wallsten T. S., & Zauberman G. (2000). The effects of averaging subjective probability estimates between and within judges. Journal of Experimental Psychology: Applied, 6(2), 130–147. 10.1037/1076-898X.6.2.130 10937317

[ref6] Attneave F. (1953). Psychological probability as a function of experienced frequency. Journal of Experimental Psychology, 46(2), 81–86. 10.1037/h0057955 13084849

[ref7] Baker C. L., Goodman N. D., & Tenenbaum J. B. (2008). Theory-based social goal inference. In Love B. C., McRae K., & Sloutsky V. M. (Eds.), Proceedings of the thirtieth annual conference of the cognitive science society (pp. 1447–1452). Cognitive Science Society.

[ref8] Baker C. L., Saxe R., & Tenenbaum J. B. (2009). Action understanding as inverse planning. Cognition, 113(3), 329–349. 10.1016/j.cognition.2009.07.005 19729154

[ref9] Baranski J. V., & Petrusic W. M. (1998). Probing the locus of confidence judgments: Experiments on the time to determine confidence. Journal of Experimental Psychology: Human Perception and Performance, 24(3), 929–945. 10.1037/0096-1523.24.3.929 9627426

[ref10] Bardolet D., Fox C. R., & Lovallo D. (2011). Corporate capital allocation: A behavioral perspective. Strategic Management Journal, 32(13), 1465–1483. 10.1002/smj.966

[ref11] Battaglia P. W., Hamrick J. B., & Tenenbaum J. B. (2013). Simulation as an engine of physical scene understanding. Proceedings of the National Academy of Sciences of the United States of America, 110(45), 18327–18332. 10.1073/pnas.1306572110 24145417 PMC3831455

[ref12] Bearden J. N., Wallsten T. S., & Fox C. R. (2007). Contrasting stochastic and support theory accounts of subadditivity. Journal of Mathematical Psychology, 51(4), 229–241. 10.1016/j.jmp.2007.04.001

[ref13] Bhui R., Lai L., & Gershman S. J. (2021). Resource-rational decision making. Current Opinion in Behavioral Sciences, 41, 15–21. 10.1016/j.cobeha.2021.02.015

[ref14] Blurton S. P., Kyllingsbæk S., Nielsen C. S., & Bundesen C. (2020). A Poisson random walk model of response times. Psychological Review, 127(3), 362–411. 10.1037/rev0000179 32223285

[ref15] Bogacz R., Brown E., Moehlis J., Holmes P., & Cohen J. D. (2006). The physics of optimal decision making: A formal analysis of models of performance in two-alternative forced-choice tasks. Psychological Review, 113(4), 700–765. 10.1037/0033-295X.113.4.700 17014301

[ref16] Bramley N. R., Dayan P., Griffiths T. L., & Lagnado D. A. (2017). Formalizing Neurath’s ship: Approximate algorithms for online causal learning. Psychological Review, 124(3), 301–338. 10.1037/rev0000061 28240922

[ref17] Brenner L. A. (2003). A random support model of the calibration of subjective probabilities. Organizational Behavior and Human Decision Processes, 90(1), 87–110. 10.1016/S0749-5978(03)00004-9

[ref18] Brooks S., Gelman A., Jones G., & Meng X. L. (Eds.). (2011). Handbook of Markov chain Monte Carlo. CRC Press. 10.1201/b10905

[ref19] Brown S., Steyvers M., & Wagenmakers E. J. (2009). Observing evidence accumulation during multi-alternative decisions. Journal of Mathematical Psychology, 53(6), 453–462. 10.1016/j.jmp.2009.09.002

[ref20] Bundesen C. (1990). A theory of visual attention. Psychological Review, 97(4), 523–547. 10.1037/0033-295X.97.4.523 2247540

[ref21] Busemeyer J. R., Pothos E. M., Franco R., & Trueblood J. S. (2011). A quantum theoretical explanation for probability judgment errors. Psychological Review, 118(2), 193–218. 10.1037/a0022542 21480739

[ref22] Busemeyer J. R., & Townsend J. T. (1993). Decision field theory: A dynamic-cognitive approach to decision making in an uncertain environment. Psychological Review, 100(3), 432–459. 10.1037/0033-295X.100.3.432 8356185

[ref23] Calder-Travis J., Charles L., Bogacz R., & Yeung N. (2020). Bayesian confidence in optimal decisions. PsyArXiv. 10.31234/osf.io/j8sxz PMC7617410 39023934

[ref24] Chater N., & Manning C. D. (2006). Probabilistic models of language processing and acquisition. Trends in Cognitive Sciences, 10(7), 335–344. 10.1016/j.tics.2006.05.006 16784883

[ref25] Chater N., Zhu J. Q., Spicer J., Sundh J., León-Villagrá P., & Sanborn A. (2020). Probabilistic biases meet the Bayesian brain. Current Directions in Psychological Science, 29(5), 506–512. 10.1177/0963721420954801

[ref26] Chen M. H., Ibrahim J. G., & Shao Q. M. (2000). Power prior distributions for generalized linear models. Journal of Statistical Planning and Inference, 84(1–2), 121–137. 10.1016/S0378-3758(99)00140-8

[ref27] Converse B. A., & Dennis P. J. (2018). The role of “Prominent Numbers” in open numerical judgment: Strained decision makers choose from a limited set of accessible numbers. Organizational Behavior and Human Decision Processes, 147, 94–107. 10.1016/j.obhdp.2018.05.007

[ref28] Costello F., & Watts P. (2014). Surprisingly rational: Probability theory plus noise explains biases in judgment. Psychological Review, 121(3), 463–480. 10.1037/a0037010 25090427

[ref29] Costello F., & Watts P. (2017). Explaining high conjunction fallacy rates: The probability theory plus noise account. Journal of Behavioral Decision Making, 30(2), 304–321. 10.1002/bdm.1936

[ref30] Costello F., & Watts P. (2018). Invariants in probabilistic reasoning. Cognitive Psychology, 100, 1–16. 10.1016/j.cogpsych.2017.11.003 29220640

[ref31] Costello F., Watts P., & Fisher C. (2018). Surprising rationality in probability judgment: Assessing two competing models. Cognition, 170, 280–297. 10.1016/j.cognition.2017.08.012 29096329

[ref32] Courville A. C., & Daw N. (2007). The rat as particle filter. In Platt J., Koller D., Singer Y., & Roweis S. (Eds.), Advances in neural information processing systems (Vol. 20). MIT Press.

[ref33] Dasgupta I., Schulz E., & Gershman S. J. (2017). Where do hypotheses come from? Cognitive Psychology, 96, 1–25. 10.1016/j.cogpsych.2017.05.001 28586634

[ref34] Diaconis P., & Ylvisaker D. (1979). Conjugate priors for exponential families. Annals of Statistics, 7(2), 269–281. 10.1214/aos/1176344611

[ref35] Diederich A., & Oswald P. (2016). Multi-stage sequential sampling models with finite or infinite time horizon and variable boundaries. Journal of Mathematical Psychology, 74, 128–145. 10.1016/j.jmp.2016.02.010

[ref36] Dougherty M. R. P. (2001). Integration of the ecological and error models of overconfidence using a multiple-trace memory model. Journal of Experimental Psychology: General, 130(4), 579–599. 10.1037/0096-3445.130.4.579 11757870

[ref37] Dragalin V. P., Tartakovsky A. G., & Veeravalli V. V. (1999). Multihypothesis sequential probability ratio tests. I. Asymptotic optimality. IEEE Transactions on Information Theory, 45(7), 2448–2461. 10.1109/18.796383

[ref38] Dragalin V. P., Tartakovsky A. G., & Veeravalli V. V. (2000). Multihypothesis sequential probability ratio tests. II. Accurate asymptotic expansions for the expected sample size. IEEE Transactions on Information Theory, 46(4), 1366–1383. 10.1109/18.850677

[ref39] Drugowitsch J., Mendonça A. G., Mainen Z. F., & Pouget A. (2019). Learning optimal decisions with confidence. Proceedings of the National Academy of Sciences of the United States of America, 116(49), 24872–24880. 10.1073/pnas.1906787116 31732671 PMC6900530

[ref40] Drugowitsch J., Moreno-Bote R., Churchland A. K., Shadlen M. N., & Pouget A. (2012). The cost of accumulating evidence in perceptual decision making. The Journal of Neuroscience, 32(11), 3612–3628. 10.1523/JNEUROSCI.4010-11.2012 22423085 PMC3329788

[ref41] Drugowitsch J., Wyart V., Devauchelle A. D., & Koechlin E. (2016). Computational precision of mental inference as critical source of human choice suboptimality. Neuron, 92(6), 1398–1411. 10.1016/j.neuron.2016.11.005 27916454

[ref42] Edwards W. (1965). Optimal strategies for seeking information—Models for statistics, choice reaction-times, and human information-processing. Journal of Mathematical Psychology, 2(2), 312–329. 10.1016/0022-2496(65)90007-6

[ref43] Epley N., & Gilovich T. (2006). The anchoring-and-adjustment heuristic: Why the adjustments are insufficient. Psychological Science, 17(4), 311–318. 10.1111/j.1467-9280.2006.01704.x 16623688

[ref44] Erev I., Wallsten T. S., & Budescu D. V. (1994). Simultaneous over-and underconfidence: The role of error in judgment processes. Psychological Review, 101(3), 519–527. 10.1037/0033-295X.101.3.519

[ref45] Fantino E., Kulik J., Stolarz-Fantino S., & Wright W. (1997). The conjunction fallacy: A test of averaging hypotheses. Psychonomic Bulletin & Review, 4(1), 96–101. 10.3758/BF03210779

[ref46] Fehr E., & Rangel A. (2011). Neuroeconomic foundations of economic choice—Recent advances. The Journal of Economic Perspectives, 25(4), 3–30. 10.1257/jep.25.4.321595323

[ref47] Feller W. (1968). An introduction to probability theory and its applications (Vol. 1). Wiley.

[ref48] Fiedler K. (1991). Heuristics and biases in theory formation: On the cognitive processes of those concerned with cognitive processes. Theory & Psychology, 1(4), 407–430. 10.1177/0959354391014002

[ref49] Findling C., & Wyart V. (2021). Computation noise in human learning and decision-making: Origin, impact, function. Current Opinion in Behavioral Sciences, 38, 124–132. 10.1016/j.cobeha.2021.02.018

[ref50] Fischhoff B., Slovic P., & Lichtenstein S. (1978). Fault trees: Sensitivity of estimated failure probabilities to problem representation. Journal of Experimental Psychology: Human Perception and Performance, 4(2), 330–344. 10.1037/0096-1523.4.2.330

[ref51] Fiser J., Berkes P., Orbán G., & Lengyel M. (2010). Statistically optimal perception and learning: From behavior to neural representations. Trends in Cognitive Sciences, 14(3), 119–130. 10.1016/j.tics.2010.01.003 20153683 PMC2939867

[ref52] Fisk J. E., & Pidgeon N. (1996). Component probabilities and the conjunction fallacy: Resolving signed summation and the low component model in a contingent approach. Acta Psychologica, 94(1), 1–20. 10.1016/0001-6918(95)00048-8

[ref53] Fleming S. M., & Lau H. C. (2014). How to measure metacognition. Frontiers in Human Neuroscience, 8, Article 443. 10.3389/fnhum.2014.00443 25076880 PMC4097944

[ref54] Fox C. R., & Rottenstreich Y. (2003). Partition priming in judgment under uncertainty. Psychological Science, 14(3), 195–200. 10.1111/1467-9280.02431 12741740

[ref55] Fränken J., Theodoropoulos N. C., & Bramley N. R. (2021). Algorithms of Adaptation in Inductive Inference. PsyArXiv. 10.31234/osf.io/ysndt 35872374

[ref56] Gardner M. (1978). Mathematical games. Scientific American, 239(2), 18–25. 10.1038/scientificamerican0878-18

[ref57] Garrett H. E. (1922). A study of the relation of accuracy to speed. Nabu Press.

[ref58] Gelman A., Stern H. S., Carlin J. B., Dunson D. B., Vehtari A., & Rubin D. B. (2013). Bayesian data analysis. Chapman & Hall. 10.1201/b16018

[ref59] Gershman S. J., & Beck J. M. (2017). Complex probabilistic inference: From cognition to neural computation. In Mustafa A. (Ed.), Computational models of brain and behavior (pp. 453–466). Wiley-Blackwell.

[ref60] Gershman S. J., Blei D. M., & Niv Y. (2010). Context, learning, and extinction. Psychological Review, 117(1), 197–209. 10.1037/a0017808 20063968

[ref61] Geyer C. (1991). Markov chain Monte Carlo maximum likelihood. In Kaufman S. M. (Ed.), Computing science and statistics: Proceedings of 23rd symposium on the interface (pp. 156–163). Interface foundation.

[ref62] Gilden D. L. (2001). Cognitive emissions of 1/*f* noise. Psychological Review, 108(1), 33–56. 10.1037/0033-295X.108.1.33 11212631

[ref63] Gilden D. L., Thornton T., & Mallon M. W. (1995). 1/*f* noise in human cognition. Science, 267(5205), 1837–1839. 10.1126/science.7892611 7892611

[ref64] Gold J. I., & Shadlen M. N. (2002). Banburismus and the brain: Decoding the relationship between sensory stimuli, decisions, and reward. Neuron, 36(2), 299–308. 10.1016/S0896-6273(02)00971-6 12383783

[ref65] Gold J. I., & Shadlen M. N. (2007). The neural basis of decision making. Annual Review of Neuroscience, 30(1), 535–574. 10.1146/annurev.neuro.29.051605.113038 17600525

[ref66] Green D. M., & Swets J. A. (1966). Signal detection theory and psychophysics. Wiley.

[ref67] Griffiths T. L., Vul E., & Sanborn A. N. (2012). Bridging levels of analysis for probabilistic models of cognition. Current Directions in Psychological Science, 21(4), 263–268. 10.1177/0963721412447619

[ref68] Haefner R. M., Berkes P., & Fiser J. (2016). Perceptual decision-making as probabilistic inference by neural sampling. Neuron, 90(3), 649–660. 10.1016/j.neuron.2016.03.020 27146267

[ref69] Hamrick J. B., Smith K. A., Griffiths T. L., & Vul E. (2015). Think again? The amount of mental simulation tracks uncertainty in the outcome. In Noelle D. C., Dale R., Warlaumont A. S., Yoshimi J., Matlock T., Jennings C. D., & Maglio P. P. (Eds.), Proceedings of the thirtieth annual conference of the cognitive science society (pp. 866–871). Cognitive Science Society.

[ref70] Hawkins G. E., & Heathcote A. (2021). Racing against the clock: Evidence-based versus time-based decisions. Psychological Review, 128(2), 222–263. 10.1037/rev0000259 33600202

[ref71] Heathcote A., Brown S., & Mewhort D. J. K. (2002). Quantile maximum likelihood estimation of response time distributions. Psychonomic Bulletin & Review, 9(2), 394–401. 10.3758/BF03196299 12120806

[ref72] Henmon V. A. C. (1911). The relation of the time of a judgment to its accuracy. Psychological Review, 18(3), 186–201. 10.1037/h0074579

[ref73] Hick W. E. (1952). On the rate of gain of information. The Quarterly Journal of Experimental Psychology, 4(1), 11–26. 10.1080/17470215208416600

[ref74] Hilbert M. (2012). Toward a synthesis of cognitive biases: How noisy information processing can bias human decision making. Psychological Bulletin, 138(2), 211–237. 10.1037/a0025940 22122235

[ref75] Honda H., Kagawa R., & Shirasuna M. (2022). On the round number bias and wisdom of crowds in different response formats for numerical estimation. Scientific Reports, 12(1), Article 8167. 10.1038/s41598-022-11900-7 35581220 PMC9114128

[ref76] Hoyer P. O., & Hyvärinen A. (2003). Interpreting neural response variability as Monte Carlo sampling of the posterior [Conference session]. Advances in Neural Information Processing Systems.

[ref77] Ibrahim J. G., Chen M. H., Gwon Y., & Chen F. (2015). The power prior: Theory and applications. Statistics in Medicine, 34(28), 3724–3749. 10.1002/sim.6728 26346180 PMC4626399

[ref78] Irwin F. W., Smith W. A. S., & Mayfield J. F. (1956). Tests of two theories of decision in an expanded judgment situation. Journal of Experimental Psychology, 51(4), 261–268. 10.1037/h0041911 13306875

[ref79] Jazayeri M., & Movshon J. A. (2007). A new perceptual illusion reveals mechanisms of sensory decoding. Nature, 446(7138), 912–915. 10.1038/nature05739 17410125 PMC3010210

[ref80] Jerez-Fernandez A., Angulo A. N., & Oppenheimer D. M. (2014). Show me the numbers: Precision as a cue to others’ confidence. Psychological Science, 25(2), 633–635. 10.1177/0956797613504301 24317423

[ref81] Johnson D. M. (1939). Confidence and speed in the two-category judgment. Archives de Psychologie, 34, 1–53.

[ref82] Juslin P., & Olsson H. (1997). Thurstonian and Brunswikian origins of uncertainty in judgment: A sampling model of confidence in sensory discrimination. Psychological Review, 104(2), 344–366. 10.1037/0033-295X.104.2.344 9162950

[ref83] Juslin P., & Persson M. (2002). PROBabilities from EXemplars (PROBEX): A “lazy” algorithm for probabilistic inference from generic knowledge. Cognitive Science, 26(5), 563–607. 10.1207/s15516709cog2605_2

[ref84] Juslin P., Winman A., & Hansson P. (2007). The naïve intuitive statistician: A naïve sampling model of intuitive confidence intervals. Psychological Review, 114(3), 678–703. 10.1037/0033-295X.114.3.678 17638502

[ref85] Juslin P., Winman A., & Olsson H. (2003). Calibration, additivity, and source independence of probability judgments in general knowledge and sensory discrimination tasks. Organizational Behavior and Human Decision Processes, 92(1–2), 34–51. 10.1016/S0749-5978(03)00063-3

[ref86] Kellen D., Winiger S., Dunn J. C., & Singmann H. (2021). Testing the foundations of signal detection theory in recognition memory. Psychological Review, 128(6), 1022–1050. 10.1037/rev0000288 34110843

[ref87] Kepecs A., & Mainen Z. F. (2012). A computational framework for the study of confidence in humans and animals. Philosophical Transactions of the Royal Society B, 367(1594), 1322–1337. 10.1098/rstb.2012.0037 PMC3318772 22492750

[ref88] Kiani R., Corthell L., & Shadlen M. N. (2014). Choice certainty is informed by both evidence and decision time. Neuron, 84(6), 1329–1342. 10.1016/j.neuron.2014.12.015 25521381 PMC4271191

[ref89] Körding K. P., & Wolpert D. M. (2004). Bayesian integration in sensorimotor learning. Nature, 427(6971), 244–247. 10.1038/nature02169 14724638

[ref90] Krajbich I., Armel C., & Rangel A. (2010). Visual fixations and the computation and comparison of value in simple choice. Nature Neuroscience, 13(10), 1292–1298. 10.1038/nn.2635 20835253

[ref91] Krajbich I., & Rangel A. (2011). Multialternative drift-diffusion model predicts the relationship between visual fixations and choice in value-based decisions. Proceedings of the National Academy of Sciences of the United States of America, 108(33), 13852–13857. 10.1073/pnas.1101328108 21808009 PMC3158210

[ref92] Kvam P. D. (2019). A geometric framework for modeling dynamic decisions among arbitrarily many alternatives. Journal of Mathematical Psychology, 91, 14–37. 10.1016/j.jmp.2019.03.001

[ref93] Kvam P. D., & Busemeyer J. R. (2020). A distributional and dynamic theory of pricing and preference. Psychological Review, 127(6), 1053–1078. 10.1037/rev0000215 32463254 PMC8407094

[ref94] Kvam P. D., Marley A. A. J., & Heathcote A. (2022). A unified theory of discrete and continuous responding. Psychological Review. Advance online publication. 10.1037/rev0000378 35862077

[ref95] Kvam P. D., & Turner B. M. (2021). Reconciling similarity across models of continuous selections. Psychological Review, 128(4), 766–786. 10.1037/rev0000296 34081510

[ref96] Kwisthout J., Wareham T., & van Rooij I. (2011). Bayesian intractability is not an ailment that approximation can cure. Cognitive Science, 35(5), 779–784. 10.1111/j.1551-6709.2011.01182.x 21609357

[ref97] Lake B. M., Ullman T. D., Tenenbaum J. B., & Gershman S. J. (2017). Building machines that learn and think like people. Behavioral and Brain Sciences, 40, Article e253. 10.1017/S0140525X16001837 27881212

[ref98] Laming D. R. J. (1968). Information theory of choice-reaction times. Academic Press.

[ref99] Lengyel M., Koblinger Á., Popović M., & Fiser J. (2015). On the role of time in perceptual decision making. arXiv preprint arXiv:1502.03135.

[ref100] Levy R., Reali F., & Griffiths T. (2008). Modeling the effects of memory on human online sentence processing with particle filters [Conference session]. Advances in Neural Information Processing Systems.

[ref101] Li H. H., & Ma W. J. (2020). Confidence reports in decision-making with multiple alternatives violate the Bayesian confidence hypothesis. Nature Communications, 11(1), Article 2004. 10.1038/s41467-020-15581-6 PMC7181620 32332712

[ref102] Lieder F., & Griffiths T. L. (2020). Resource-rational analysis: Understanding human cognition as the optimal use of limited computational resources. Behavioral and Brain Sciences, 43, Article E1. 10.1017/S0140525X1900061X 30714890

[ref103] Lieder F., Griffiths T. L., Huys Q. J. M., & Goodman N. D. (2018). The anchoring bias reflects rational use of cognitive resources. Psychonomic Bulletin & Review, 25(1), 322–349. 10.3758/s13423-017-1286-8 28484952

[ref104] Link S. W., & Heath R. A. (1975). A sequential theory of psychological discrimination. Psychometrika, 40(1), 77–105. 10.1007/BF02291481

[ref105] Linkenkaer-Hansen K., Nikouline V. V., Palva J. M., & Ilmoniemi R. J. (2001). Long-range temporal correlations and scaling behavior in human brain oscillations. The Journal of Neuroscience, 21(4), 1370–1377. 10.1523/JNEUROSCI.21-04-01370.2001 11160408 PMC6762238

[ref106] Logan G. D. (1988). Toward an instance theory of automatization. Psychological Review, 95(4), 492–527. 10.1037/0033-295X.95.4.492

[ref107] Luce R. D. (1986). Response times: Their role in inferring elementary mental organization (No. 8). Oxford University Press on Demand.

[ref108] Luu L., & Stocker A. A. (2018). Post-decision biases reveal a self-consistency principle in perceptual inference. eLife, 7, Article e33334. 10.7554/eLife.33334 29785928 PMC5963926

[ref109] Ma Y. A., Chen Y., Jin C., Flammarion N., & Jordan M. I. (2019). Sampling can be faster than optimization. Proceedings of the National Academy of Sciences of the United States of America, 116(42), 20881–20885. 10.1073/pnas.1820003116 31570618 PMC6800351

[ref110] Mamassian P. (2016). Visual confidence. Annual Review of Vision Science, 2(1), 459–481. 10.1146/annurev-vision-111815-114630 28532359

[ref111] Maniscalco B., & Lau H. (2012). A signal detection theoretic approach for estimating metacognitive sensitivity from confidence ratings. Consciousness and Cognition, 21(1), 422–430. 10.1016/j.concog.2011.09.021 22071269

[ref112] Marr D. (1982). Vision: A computational investigation into the human representation and processing of visual information. W. H. Freeman.

[ref113] McCarley J. S., & Benjamin A. S. (2013). Bayesian and signal detection models. In Lee J. D. & Kirlik A. (Eds.), The Oxford handbook of cognitive engineering (pp. 465–475). Oxford University Press. 10.1093/oxfordhb/9780199757183.013.0032

[ref114] Medin D. L., & Schaffer M. M. (1978). Context theory of classification learning. Psychological Review, 85(3), 207–238. 10.1037/0033-295X.85.3.207

[ref115] Mehle T., Gettys C. F., Manning C., Baca S., & Fisher S. (1981). The availability explanation of excessive plausibility assessments. Acta Psychologica, 49(2), 127–140. 10.1016/0001-6918(81)90024-X

[ref116] Miletić S., Boag R. J., Trutti A. C., Stevenson N., Forstmann B. U., & Heathcote A. (2021). A new model of decision processing in instrumental learning tasks. eLife, 10, Article e63055. 10.7554/eLife.63055 33501916 PMC7880686

[ref117] Milosavljevic M., Malmaud J., Huth A., Koch C., & Rangel A. (2010). The drift diffusion model can account for value-based choice response times under high and low time pressure. Judgment and Decision Making, 5(6), 437–449. 10.1017/S1930297500001285

[ref118] Moran R. (2015). Optimal decision making in heterogeneous and biased environments. Psychonomic Bulletin & Review, 22(1), 38–53. 10.3758/s13423-014-0669-3 24928091

[ref119] Nilsson H., Juslin P., & Winman A. (2016). Heuristics can produce surprisingly rational probability estimates: Comment on Costello and Watts (2014). Psychological Review, 123(1), 103–111. 10.1037/a0039249 26709414

[ref120] Nilsson H., Winman A., Juslin P., & Hansson G. (2009). Linda is not a bearded lady: Configural weighting and adding as the cause of extension errors. Journal of Experimental Psychology: General, 138(4), 517–534. 10.1037/a0017351 19883134

[ref121] Norris D. (2006). The Bayesian reader: Explaining word recognition as an optimal Bayesian decision process. Psychological Review, 113(2), 327–357. 10.1037/0033-295X.113.2.327 16637764

[ref122] Nosofsky R. M. (1984). Choice, similarity, and the context theory of classification. Journal of Experimental Psychology: Learning, Memory, and Cognition, 10(1), 104–114. 10.1037/0278-7393.10.1.104 6242730

[ref123] Nosofsky R. M. (1986). Attention, similarity, and the identification-categorization relationship. Journal of Experimental Psychology: General, 115(1), 39–61. 10.1037/0096-3445.115.1.39 2937873

[ref124] Nosofsky R. M., & Palmeri T. J. (1997). An exemplar-based random walk model of speeded classification. Psychological Review, 104(2), 266–300. 10.1037/0033-295X.104.2.266 9127583

[ref125] Novikov E., Novikov A., Shannahoff-Khalsa D., Schwartz B., & Wright J. (1997). Scale-similar activity in the brain. Physical Review E: Statistical Physics, Plasmas, Fluids, and Related Interdisciplinary Topics, 56(3), R2387–R2389. 10.1103/PhysRevE.56.R2387

[ref126] Olson C. L. (1976). Some apparent violations of the representativeness heuristic in human judgment. Journal of Experimental Psychology: Human Perception and Performance, 2(4), 599–608. 10.1037/0096-1523.2.4.599

[ref127] Pachella R. G. (1974). The interpretation of reaction time in information-processing research. In Kantowitz B. H. (Ed.), Human information processing: Tutorials in performance and cognition (pp. 41–82). Lawrence Erlbaum.

[ref128] Peterson C. R., & Beach L. R. (1967). Man as an intuitive statistician. Psychological Bulletin, 68(1), 29–46. 10.1037/h0024722 6046307

[ref129] Pleskac T. J., & Busemeyer J. R. (2010). Two-stage dynamic signal detection: A theory of choice, decision time, and confidence. Psychological Review, 117(3), 864–901. 10.1037/a0019737 20658856

[ref130] Pothos E. M., & Busemeyer J. R. (2022). Quantum cognition. Annual Review of Psychology, 73(1), 749–778. 10.1146/annurev-psych-033020-123501 34546804

[ref131] Pouget A., Drugowitsch J., & Kepecs A. (2016). Confidence and certainty: Distinct probabilistic quantities for different goals. Nature Neuroscience, 19(3), 366–374. 10.1038/nn.4240 26906503 PMC5378479

[ref132] Proctor R. W., & Schneider D. W. (2018). Hick’s law for choice reaction time: A review. Quarterly Journal of Experimental Psychology, 71(6), 1281–1299. 10.1080/17470218.2017.1322622 28434379

[ref133] Rasmussen C. E., & Ghahramani Z. (2002). Bayesian Monte Carlo [Conference session]. Advances in Neural Information Processing Systems.

[ref134] Ratcliff R. (1978). A theory of memory retrieval. Psychological Review, 85(2), 59–108. 10.1037/0033-295X.85.2.59

[ref135] Ratcliff R. (1981). A theory of order relations in perceptual matching. Psychological Review, 88(6), 552–572. 10.1037/0033-295X.88.6.552

[ref136] Ratcliff R. (2018). Decision making on spatially continuous scales. Psychological Review, 125(6), 888–935. 10.1037/rev0000117 30431302 PMC6242349

[ref137] Ratcliff R., Gomez P., & McKoon G. (2004). A diffusion model account of the lexical decision task. Psychological Review, 111(1), 159–182. 10.1037/0033-295X.111.1.159 14756592 PMC1403837

[ref138] Ratcliff R., & McKoon G. (2008). The diffusion decision model: Theory and data for two-choice decision tasks. Neural Computation, 20(4), 873–922. 10.1162/neco.2008.12-06-420 18085991 PMC2474742

[ref139] Ratcliff R., & Rouder J. N. (1998). Modeling response times for two-choice decisions. Psychological Science, 9(5), 347–356. 10.1111/1467-9280.00067

[ref140] Ratcliff R., & Smith P. (2015). Modeling simple decisions and applications using a diffusion model. In Busemeyer J. R., Wang Z., Townsend J. T., & Eidels A. (Eds.), The Oxford handbook of computational and mathematical psychology (pp. 35–62). Oxford University Press.

[ref141] Ratcliff R., Smith P. L., Brown S. D., & McKoon G. (2016). Diffusion decision model: Current issues and history. Trends in Cognitive Sciences, 20(4), 260–281. 10.1016/j.tics.2016.01.007 26952739 PMC4928591

[ref142] Ratcliff R., Smith P. L., & McKoon G. (2015). Modeling regularities in response time and accuracy data with the diffusion model. Current Directions in Psychological Science, 24(6), 458–470. 10.1177/0963721415596228 26722193 PMC4692464

[ref143] Ratcliff R., & Starns J. J. (2009). Modeling confidence and response time in recognition memory. Psychological Review, 116(1), 59–83. 10.1037/a0014086 19159148 PMC2693899

[ref144] Ratcliff R., Thapar A., & McKoon G. (2003). A diffusion model analysis of the effects of aging on brightness discrimination. Perception & Psychophysics, 65(4), 523–535. 10.3758/BF03194580 12812276 PMC1360154

[ref145] Ratcliff R., Thapar A., & McKoon G. (2011). Effects of aging and IQ on item and associative memory. Journal of Experimental Psychology: General, 140(3), 464–487. 10.1037/a0023810 21707207 PMC3149731

[ref146] Robert C. P., & Casella G. (2004). Monte Carlo statistical methods (Vol. 2). Springer. 10.1007/978-1-4757-4145-2

[ref147] Roberts G. O., & Stramer O. (2002). Langevin diffusions and Metropolis-Hastings algorithms. Methodology and Computing in Applied Probability, 4(4), 337–357. 10.1023/A:1023562417138

[ref148] Rossky P. J., Doll J. D., & Friedman H. L. (1978). Brownian dynamics as smart Monte Carlo simulation. The Journal of Chemical Physics, 69(10), 4628–4633. 10.1063/1.436415

[ref149] Rotello C. M. (2018). Signal detection theories of recognition memory. In Byrne J. H. & Wixted J. T. (Eds.), Learning and memory: A comprehensive reference, Vol. 2: Cognitive psychology of memory (2nd ed., pp. 201–226). Elsevier.

[ref150] Rouder J. N. (1996). Premature sampling in random walks. Journal of Mathematical Psychology, 40(4), 289–296. 10.1006/jmps.1996.0030

[ref151] Russo J. E., & Kolzow K. J. (1994). Where is the fault in fault trees? Journal of Experimental Psychology: Human Perception and Performance, 20(1), 17–32. 10.1037/0096-1523.20.1.17

[ref152] Sanborn A. N. (2017). Types of approximation for probabilistic cognition: Sampling and variational. Brain and Cognition, 112, 98–101. 10.1016/j.bandc.2015.06.008 26228974

[ref153] Sanborn A. N., & Beierholm U. R. (2016). Fast and accurate learning when making discrete numerical estimates. PLOS Computational Biology, 12(4), Article e1004859. 10.1371/journal.pcbi.1004859 27070155 PMC4829178

[ref154] Sanborn A. N., & Chater N. (2016). Bayesian brains without probabilities. Trends in Cognitive Sciences, 20(12), 883–893. 10.1016/j.tics.2016.10.003 28327290

[ref155] Sanborn A. N., Griffiths T. L., & Navarro D. J. (2010). Rational approximations to rational models: Alternative algorithms for category learning. Psychological Review, 117(4), 1144–1167. 10.1037/a0020511 21038975

[ref156] Sanborn A. N., Mansinghka V. K., & Griffiths T. L. (2013). Reconciling intuitive physics and Newtonian mechanics for colliding objects. Psychological Review, 120(2), 411–437. 10.1037/a0031912 23458084

[ref157] See K. E., Fox C. R., & Rottenstreich Y. S. (2006). Between ignorance and truth: Partition dependence and learning in judgment under uncertainty. Journal of Experimental Psychology: Learning, Memory, and Cognition, 32(6), 1385–1402. 10.1037/0278-7393.32.6.1385 17087591

[ref158] Shekhar M., & Rahnev D. (2021a). The nature of metacognitive inefficiency in perceptual decision making. Psychological Review, 128(1), 45–70. 10.1037/rev0000249 32673034 PMC7883626

[ref159] Shekhar M., & Rahnev D. (2021b). Sources of metacognitive inefficiency. Trends in Cognitive Sciences, 25(1), 12–23. 10.1016/j.tics.2020.10.007 33214066 PMC8610081

[ref160] Shepard R. N. (1987). Toward a universal law of generalization for psychological science. Science, 237(4820), 1317–1323. 10.1126/science.3629243 3629243

[ref161] Sheu C. F., & Ratcliff R. (1995). The application of Fourier deconvolution to reaction time data: A cautionary note. Psychological Bulletin, 118(2), 285–299. 10.1037/0033-2909.118.2.285 7568573

[ref162] Sloman S., Rottenstreich Y., Wisniewski E., Hadjichristidis C., & Fox C. R. (2004). Typical versus atypical unpacking and superadditive probability judgment. Journal of Experimental Psychology: Learning, Memory, and Cognition, 30(3), 573–582. 10.1037/0278-7393.30.3.573 15099126

[ref163] Smith P. L. (2016). Diffusion theory of decision making in continuous report. Psychological Review, 123(4), 425–451. 10.1037/rev0000023 26949831

[ref164] Spicer J., Zhu J.-Q., Chater N., & Sanborn A. N. (2022a). Perceptual and cognitive judgments show both anchoring and repulsion. Psychological Science, 33(9), 1395–1407. 10.1177/09567976221089599 35876741

[ref165] Spicer J., Zhu J.-Q., Chater N., & Sanborn A. N. (2022b). How do people predict a random walk? Lessons for models of human cognition. PsyArXiv. 10.31234/osf.io/fjtha 39298225

[ref166] Starns J. J., & Ratcliff R. (2014). Validating the unequal-variance assumption in recognition memory using response time distributions instead of ROC functions: A diffusion model analysis. Journal of Memory and Language, 70, 36–52. 10.1016/j.jml.2013.09.005 24459327 PMC3896247

[ref167] Stengård E., & van den Berg R. (2019). Imperfect Bayesian inference in visual perception. PLOS Computational Biology, 15(4), Article e1006465. 10.1371/journal.pcbi.1006465 30998675 PMC6472731

[ref168] Stone M. (1960). Models for choice-reaction time. Psychometrika, 25(3), 251–260. 10.1007/BF02289729

[ref169] Strack F., & Mussweiler T. (1997). Explaining the enigmatic anchoring effect: Mechanisms of selective accessibility. Journal of Personality and Social Psychology, 73(3), 437–446. 10.1037/0022-3514.73.3.437

[ref170] Sundh J., Zhu J.-Q., Chater N., & Sanborn A. N. (2021). The mean-variance signature of Bayesian probability judgment. PsyArXiv. 10.31234/osf.io/yuhaz

[ref171] Swensson R. G. (1972). The elusive tradeoff: Speed vs accuracy in visual discrimination tasks. Perception & Psychophysics, 12(1), 16–32. 10.3758/BF03212837

[ref172] Tauber S., Navarro D. J., Perfors A., & Steyvers M. (2017). Bayesian models of cognition revisited: Setting optimality aside and letting data drive psychological theory. Psychological Review, 124(4), 410–441. 10.1037/rev0000052 28358549

[ref173] Tentori K., Crupi V., & Russo S. (2013). On the determinants of the conjunction fallacy: Probability versus inductive confirmation. Journal of Experimental Psychology: General, 142(1), 235–255. 10.1037/a0028770 22823498

[ref174] Tian L., Ellis K., Kryven M., & Tenenbaum J. (2020). Learning abstract structure for drawing by efficient motor program induction [Conference session]. Advances in Neural Information Processing Systems.

[ref175] Tickle H., Tsetsos K., Speekenbrink M., & Summerfield C. (2023). Human optional stopping in a heteroscedastic world. Psychological Review, 130(1), 1–22. 10.1037/rev0000315 34570524

[ref176] Townsend J. T., & Ashby F. G. (1983). Stochastic modeling of elementary psychological processes. CUP Archive.

[ref177] Trippas D., Kellen D., Singmann H., Pennycook G., Koehler D. J., Fugelsang J. A., & Dubé C. (2018). Characterizing belief bias in syllogistic reasoning: A hierarchical Bayesian meta-analysis of ROC data. Psychonomic Bulletin & Review, 25(6), 2141–2174. 10.3758/s13423-018-1460-7 29943172 PMC6267550

[ref178] Turner B. M., & Van Zandt T. (2012). A tutorial on approximate Bayesian computation. Journal of Mathematical Psychology, 56(2), 69–85. 10.1016/j.jmp.2012.02.005

[ref179] Tversky A., & Kahneman D. (1974). Judgment under uncertainty: Heuristics and biases. Science, 185(4157), 1124–1131. 10.1126/science.185.4157.1124 17835457

[ref180] Tversky A., & Kahneman D. (1983). Extensional versus intuitive reasoning: The conjunction fallacy in probability judgment. Psychological Review, 90(4), 293–315. 10.1037/0033-295X.90.4.293

[ref181] Tversky A., & Koehler D. J. (1994). Support theory: A nonextensional representation of subjective probability. Psychological Review, 101(4), 547–567. 10.1037/0033-295X.101.4.547

[ref182] Usher M., & McClelland J. L. (2004). Loss aversion and inhibition in dynamical models of multialternative choice. Psychological Review, 111(3), 757–769. 10.1037/0033-295X.111.3.757 15250782

[ref183] Van Orden G. C., Holden J. G., & Turvey M. T. (2003). Self-organization of cognitive performance. Journal of Experimental Psychology: General, 132(3), 331–350. 10.1037/0096-3445.132.3.331 13678372

[ref184] van Ravenzwaaij D., Brown S. D., Marley A. A. J., & Heathcote A. (2020). Accumulating advantages: A new conceptualization of rapid multiple choice. Psychological Review, 127(2), 186–215. 10.1037/rev0000166 31580104

[ref185] Van Sommers P. (1984). Drawing and cognition: Descriptive and experimental studies of graphic production processes. Cambridge University Press. 10.1017/CBO9780511897672

[ref186] Van Zandt T. (2000). ROC curves and confidence judgements in recognition memory. Journal of Experimental Psychology: Learning, Memory, and Cognition, 26(3), 582–600. 10.1037/0278-7393.26.3.582 10855419

[ref187] Varey C. A., Mellers B. A., & Birnbaum M. H. (1990). Judgments of proportions. Journal of Experimental Psychology: Human Perception and Performance, 16(3), 613–625. 10.1037/0096-1523.16.3.613 2144575

[ref188] Vickers D. (1979). Decision processes in visual perception. Academic Press.

[ref189] Vickers D., & Packer J. (1982). Effects of alternating set for speed or accuracy on response time, accuracy and confidence in a unidimensional discrimination task. Acta Psychologica, 50(2), 179–197. 10.1016/0001-6918(82)90006-3 7102359

[ref190] Vul E., Goodman N., Griffiths T. L., & Tenenbaum J. B. (2014). One and done? Optimal decisions from very few samples. Cognitive Science, 38(4), 599–637. 10.1111/cogs.12101 24467492

[ref191] Wagenmakers E. J., Farrell S., & Ratcliff R. (2004). Estimation and interpretation of 1/f^α^ noise in human cognition. Psychonomic Bulletin & Review, 11(4), 579–615. 10.3758/BF03196615 15581115 PMC1479451

[ref192] Wald A. (1949). Statistical decision functions. Annals of Mathematical Statistics, 20(2), 165–205. 10.1214/aoms/1177730030

[ref193] Wald A., & Wolfowitz J. (1948). Optimum character of the sequential probability ratio test. Annals of Mathematical Statistics, 19(3), 326–339. 10.1214/aoms/1177730197

[ref194] Welsh M., Navarro D., & Begg S. (2011). Number preference, precision and implicit confidence [Paper presentation]. Proceedings of the Annual Meeting of the Cognitive Science Society, Texas, United States.

[ref195] Wickelgren W. A. (1977). Speed–accuracy tradeoff and information-processing dynamics. Acta Psychologica, 41(1), 67–85. 10.1016/0001-6918(77)90012-9

[ref196] Wyart V., & Koechlin E. (2016). Choice variability and suboptimality in uncertain environments. Current Opinion in Behavioral Sciences, 11, 109–115. 10.1016/j.cobeha.2016.07.003

[ref197] Yeung N., & Summerfield C. (2014). Shared mechanisms for confidence judgements and error detection in human decision making. In Fleming S. M. & Frith C. D. (Eds.), The cognitive neuroscience of metacognition (pp. 147–167). Springer. 10.1007/978-3-642-45190-4_7

[ref198] Yuille A., & Kersten D. (2006). Vision as Bayesian inference: Analysis by synthesis? Trends in Cognitive Sciences, 10(7), 301–308. 10.1016/j.tics.2006.05.002 16784882

[ref199] Zamboni E., Ledgeway T., McGraw P. V., & Schluppeck D. (2016). Do perceptual biases emerge early or late in visual processing? Decision-biases in motion perception. Proceedings of the Royal Society B: Biological Sciences, 283(1833), Article 20160263. 10.1098/rspb.2016.0263 PMC4936027 27335413

[ref200] Zellner A. (2002). Information processing and Bayesian analysis. Journal of Econometrics, 107(1–2), 41–50. 10.1016/S0304-4076(01)00112-9

[ref201] Zhu J.-Q., León-Villagrá P., Chater N., & Sanborn A. N. (2022). Understanding the structure of cognitive noise. PLOS Computational Biology, 18(8), Article e1010312. 10.1371/journal.pcbi.1010312 35976980 PMC9423631

[ref202] Zhu J.-Q., Newall P. W. S., Sundh J., Chater N., & Sanborn A. N. (2022). Clarifying the relationship between coherence and accuracy in probability judgments. Cognition, 223, Article 105022. 10.1016/j.cognition.2022.105022 35074619 PMC8987733

[ref203] Zhu J.-Q., Sanborn A. N., & Chater N. (2018). Mental sampling in multimodal representations [Conference session]. Advances in Neural Information Processing Systems.

[ref204] Zhu J.-Q., Sanborn A. N., & Chater N. (2019). Why decisions bias perception: An amortised sequential sampling account [Conference session]. Proceedings of Annual Meeting of the Cognitive Science Society, Texas, United States.

[ref205] Zhu J.-Q., Sanborn A. N., & Chater N. (2020). The Bayesian sampler: Generic Bayesian inference causes incoherence in human probability judgments. Psychological Review, 127(5), 719–748. 10.1037/rev0000190 32191073 PMC7571263

[ref206] Zhu J.-Q., Spicer J., Sanborn A. N., & Chater N. (2021). Cognitive variability matches speculative price dynamics. PsyArXiv. 10.31234/osf.io/gfjvs

[ref207] Zhu J.-Q., Sundh J., Spicer J., Chater N., & Sanborn A. N. (2022, October 6). The autocorrelated bayesian sampler: A rational process for probability judgments, estimates, confidence intervals, choices, confidence judgments, and response times. https://osf.io/8wp5a/10.1037/rev0000427 PMC11115360 37289507

